# A Review on *Cistus* sp.: Phytochemical and Antimicrobial Activities

**DOI:** 10.3390/plants10061214

**Published:** 2021-06-15

**Authors:** Imane Zalegh, Mohamed Akssira, Mohammed Bourhia, Fouad Mellouki, Naima Rhallabi, Ahmad Mohammad Salamatullah, Mohammed Saeed Alkaltham, Heba Khalil Alyahya, Rajaa Ait Mhand

**Affiliations:** 1 Research Unit Microbiology, Hygiene & Biomolecule, Laboratory of Virology, Microbiology, Quality & Biotechnology/Ecotoxicology and Biodiversity, FSTM, University Hassan II Casablanca, Casablanca 20000, Morocco; fouad.mellouki@fstm.ac.ma (F.M.); rallabina@yahoo.fr (N.R.); rajaamhand@yahoo.fr (R.A.M.); 2 Laboratory of Physical Chemistry & Bioorganic Chemistry, Research Unit Associated CNRST (URAC 22), FSTM, University Hassan II Casablanca, Casablanca 20000, Morocco; mohamed.akssira@fstm.ac.ma; 3 Laboratory of Chemistry, Biochemistry, Nutrition, and Environment, Faculty of Medicine and Pharmacy, University Hassan II, Casablanca 20000, Morocco; bourhiamohammed@gmail.com; 4 Department of Food Science & Nutrition, College of Food and Agricultural Sciences, King Saud University, P.O. Box 2460, Riyadh 11451, Saudi Arabia; asalamh@ksu.edu.sa (A.M.S.); malkaltham@ksu.edu.sa (M.S.A.); hkalyahya@ksu.edu.sa (H.K.A.)

**Keywords:** *Cistus* sp., phytochemicals, microorganisms, antimicrobials, multidrug resistance

## Abstract

Resistance to drugs is reaching alarming levels and is placing human health at risk. With the lack of new antimicrobials drugs, infectious diseases are becoming harder to treat. Hence, there is an increasing awareness of active phytochemicals with therapeutic functions. The tremendous research interest on the *Cistus* L. genus includes numerous plants used in traditional medicine by people living around the Mediterranean Sea, also resulted in some interesting discoveries and written literature. This review aimed at gathering scientific literature about *Cistus* species, describing phytochemical profiles and the various pharmacological activities. We also extensively reviewed the antimicrobial activities, including antiviral, antiparasitic, antifungal, and antibacterial potentials of Essential Oils (EO), raw extracts as well as isolated compounds. Mechanisms of action along with methods used are also investigated in this review. Considering the findings of the *Cistus* species extracts, this genus offers an adequate reserve of active phytochemicals since many have been used to create drugs. Therefore, this review work can serve society by providing a global view on *Cistus* L. sp. regarding pharmacological potentials and their chemical profiles.

## 1. Introduction

Nature is the source of natural remedies widely used by 80% of the world population [1]. In North America, Europe, and other developed regions, over 50% of the population has used traditional medicine at least once [2].

The World Health Organization (WHO) has a keen interest in documenting medicinal plants used by indigenous people from different parts of the world [3,4]. The use of plant derivatives as medicinal treatments gained popularity in the late 1990s [5].

Plants are a biologically and chemically diverse resource. The total plant species estimated on planet Earth is about 25,000 to 500,000 [6], and more than 10% of them are used for therapeutic purposes [7]. Around 71% of drugs have directly or indirectly been derived from natural products [8].

The extraordinary advantages of bioactive molecules as a source of biotherapeutics are beyond question [9,10]. Studies have identified more than 5000 individual phytochemicals, and this number is continuously increasing due to the introduction of new and efficient isolation and characterization techniques [11,12,13,14].

Most of the studies showed that many herbs are rich in phenols, flavonoids, tannins, vitamins, and many more phytochemicals [9,10,15,16]. Moreover, researchers in the last few years have shown an increasing interest in these compounds [13,14]. Plant-derived products have potential against illnesses with no or few secondary effects when compared to synthetic ones [9,16,17,18].

The screening of phytochemical composition in medicinal and aromatic plants plays a significant role in many areas, such as the human diet, animal feed, pharmaceuticals, fragrances, and cosmetics, etc. [19,20,21,22].

The Mediterranean basin, one of the hot spot biodiversity in the world [23,24], is rich in vegetation, including medicinal plants [25,26,27]. One example is *Cistus* L. sp., which was intensively studied in terms of medicinal properties along with its chemical composition. In this sense, this work was conducted to gather data on *Cistus* L. regarding antimicrobial potential and chemical profiles.

## 2. Genus’s Presentation

*Cistus* plants are the dominant shrubs in the Mediterranean region. Most species have a widespread distribution but there are a few narrow endemics among the family *Cistaceae* L. known as rockrose [28,29]. This family comprises several perennial medicinal plants [30], with eight genera, including the *Cistus* genus [31].

The genus *Cistus* is relatively tiny and complex because of the polymorphism and the hybridization between related species [32]. It contains about 21 species after several taxonomic re-evaluations based on molecular analysis of phylogeny [33,34].

### 2.1. Botanical Presentation

The genus is composed of pioneer species [35]. The straight branches can range from 50 to 100 cm in height. On the branches, corrugated leaves grow entangled and straightforward [36]. Plants of the genus are cross-pollinated between species. Their big visible flowers are hermaphroditic, actinomorphic, and hypogynous, with three of five sepals opposite the petals [6,29,37,38]. Petal color ranges from white to purple/dark pink depending on the subgenus, with a conspicuous dark red spot at the base of each petal present in a few species [31], which makes it a common ornamental plant [39]. Besides, they have viscid stems and leaves that produce a fragrant oleoresin [40]. Most family members are very fragrant and sweet-smelling, much appreciated in the perfume industry [41].

### 2.2. Cultural Characteristics

*Cistus* species are frequent, particularly in mountainous regions with acidic and basic soils [33]. Environmental specificity referring to substrate confers additional value to acidophilous and basophils species as predictable indicators of woodland disturbances [33]. As well as an adaptation to resist summer droughts, this genus adapts well to frequent disturbance events such as poor soils, fire-degraded soils, and grazing that destroy large forest areas [42]. These species are well known for producing large quantities of seeds that resist fires and rapidly populate in the following season [32,43,44,45].

The germination is influenced by insect pollination, attracted by the odors released by the monoterpenes produced by the flowers [35,36].

Adding the ability of *Cistus* species to colonize degraded areas, these shrubs inhibit the growth of other plants by restricting their aerial growth or by inhibiting germination of other species due to its phytotoxicity over other plants and soil [39,46,47,48].

### 2.3. Chemical Analysis

The *Cistus* species are rich in polyphenolic compounds (the main components are polyphenolic compounds, commonly known as catechins). This characteristic makes these plants able to withstand extreme conditions. The metabolism of polyphenols protects the plants against different stresses, whether biotic or abiotic [49]. This abiotic protection is due to polyphenols that can reduce nitrogen’s mobility in the soil and then allow these shrubs to grow in nitrogen-deficient soils [50].

Chemical analysis of different *Cistus* species’ tissues showed different chemical classes, including diterpenes, which are usually detected in *Cistus monspeliensis* L. and *Cistus libanotis* L. [51]. These plants are also recognized to contain multiple compounds from different chemical classes such as flavonoids, coumarins, terpene derivatives, hydrocarbons [45,52]. Many studies have reported on the phytochemicals in extracts of different *Cistus* species from several regions. The first study on the *Cistus* genus, published by R. Hegnauer, determined the constitution of diterpenes in the aerial parts of *C. monspeliensis* [53]. A few years later, this genus started to interest researchers. JDP Teresa focused studies on the aerial parts of *Cistus laurifolius* L. growing in Spain, from which new terpenes were isolated: salmantic acid and its methyl ester, salmantidiol [54], also the labdane-type diterpene 6β,8-dihydroxy-ent-13E-labden-15-oic acid (laurifolic acid) [55]. Studies continued from 1990 to 2000, focusing on diterpenes by studying others species, especially *Cistus creticus* subsp. *creticus* L. from Greece, from which several labdane-type diterpenes were identified [56,57,58,59,60]. The Essential Oil (EO) of *Cistus* species is widely studied, especially *Cistus creticus* L., *Cistus ladaniferus* L., and *C. monspeliensis*. Manoyl oxide isomers and 13-epi-manoyl oxide were the most reported diterpenes in *C. monspeliensis* [61,62,63] as well as sesquiterpene oxygenated (α-cadinol as the main compound) isolated in France from leaves [64]. *C. monspeliensisis* is also known to have a high content of terpenoids. Aerial parts of *C. ladaniferus* are another example of the richness in terpenoids (oxygenated sesquiterpenes and monoterpene hydrocarbons) among them, viridiflorol, α-pinene, ledol, bornyl acetate [63,65,66,67]. A chemoprofile of *C. creticus* was highly variable due to the variance of subsp., geographic regions, and then pedoclimatic conditions due to seasonal variations. Three classes were identified: phenylpropanoids (carvacrol [68,69,70]), carbonylic compounds (norisoprenoids representing by vitispirane I [68]), and terpenes. The latter were the most reported in this species’s oil, especially diterpene in *C. creticus* subsp. creticus. The most reported diterpene were manoyl oxide, 13-epi-manoyl oxide, drimane-7,9(11)-diene, and labdane skeletons [59,68,69,70,71], and sesquiterpene (α-cadinene, δ-cadinene) which are predominant in *Cistus creticus* subsp. *Eriocephalus* Viv. [70]. *Cistus albidus* L. was also studied for its EO composition. Conversely, in this EO diterpenes and monoterpenes were absent. Sesquiterpene derivatives were dominant with high content, the main components being α-zingiberene, α-curcumene, (E)-β-caryophyllene, α-cadinol, α-bisabolol, δ-cadinene, and germacrene D [72,73,74]. A study carried out on 15 types of *Cistus salviifolius* L. from the Crete island showed 167 compounds dominated with high percentages of sesquiterpenes (Camphor and viridiflorol as main compounds) [75]. Another study on the EO composition of 5 *Cistus* species, including *salviifolius*, showed 89 components dominated by sesquiterpenes (oxygenated ones were higher than hydrocarbons represented especially by germacrene D as a leading compound, this latter was absent in EO from Crete island). Diterpenes were also present among the EO of *C. libanotis* and *Cistus villosus* L. from Tunisia, 56 and 54 elements were isolated respectively, monoterpenes hydrocarbons characterized the first species. However, the second major class of compounds was carbonylic compounds (undecan-2-one and hexahydrofarnesylacetone were the most abundant) and hydrocarbon (heptacosane and nonacosane as principal ones) [62].

Also known as *C. creticus* and *C. villosus.* Several investigations focused on aqueous and semi-organic extracts from *Cistus incanus* L., marketed as herbal infusions and dietary supplements. Viapiana’s team analyzed by HPLC the phenolic profile of hydromethanolic and aqueous extracts of different commercial samples from different regions. The results proved that all samples were rich in phenolic acids and flavonoids, gallic acid, rutin, and the most abundant of them in all samples is isoquercetin. However, there are differences in the content of the other compounds identified depending on the extraction solvent (chlorogenic acid, ferulic acid syringic acid were not found in aqueous extracts of *C. incanus*) in addition to the growing conditions, tissue maturity, and post-harvest treatment [31]. As it is now recognized, the preparation method, the temperature used, duration, and the type of water strongly influence phenolic compounds during cooking.

Riehle has proven a significant decrease of different groups of phenolic compounds and flavonoids when brewing the commercially available *C. incanus* infusions with different water used at various conditions [76]. The profile of phenolic compounds from *C. incanus* pollen was different between nonhydrolyzed and hydrolyzed. Although the in vivo data about the metabolism of various food sources of phenolic compounds isolated after feeding are contradictory and rare, it is essential to consider data on these compounds and the changes they may undergo once in the human body [77].

The study of the two Tunisian and Italian teams represented by Marcello Nicoletti and Mariem Ben Jemia, focusing on terpenes, phenols, and glycosides of raw methanolic extract of *C. monspeliensis*, showed that diterpenes were evident and polyphenols were present in different concentrations. Flavonoids were also reported with isolation of new phenylglucoside,1-(3,5-dihydroxy-2-methylphenyl)ethanone-5-*O*-β-d-glucopyranoside, named monspelioside, which was identified for the first time from the genus *Cistus* [70].

Ben Jemia identified 36 compounds from hexane extracts of *C. monspeliensis* leaves as a part of the investigation on Tunisian medicinal plants. Top-class was represented by fatty acids and a good amount of hydrocarbons; Vitamin E was also present [41]. The teams have also studied *C. libanotis*, a complete analysis of the hexane extracts from leaves, and showed 47 constituents. Flavonoids were dominant, followed by fatty acids, monoterpene hydrocarbons, hydrocarbons, oxygenated monoterpenes, diterpenes, sesquiterpenes, oxygenated sesquiterpenes, which were present in a small amount [41]. In contrast, when they focused only on terpenes and phenols constituents, they reported the evidence of diterpenes and flavonoids in methanol extracts [78]. According to the same papers, these diterpenes were rarely reported in *C. villosus*, dominated by hydrocarbons and Vitamin E and some tannins [69,71].

The first investigation on ethanolic extracts of *C. laurifolius* aerial parts from Spain concluded the isolation of a new diterpene called laurifolic and identification of five glucosides (β-d-glucopyranosiloxyethane, 4-β-d-glucopyranosiloxyacetophenone, reseoside, 1,3-dihydroxy-5-β-d-glucopyranosiloxybenzene, 1-*O*-methyl-epi-inositol) [55]. Between 2007 and 2012, Erdem Yesilada published two papers about organic extracts of *C. laurifolius* leaves from Turkey, which reported three flavonoids (3-*O*-/methylquercetin, 3,7-*O*-dimethylquercetin, and 3,7-*O*-dimethylkaempferol) [73,74]. Considering the wide use of the *C. laurifolius* by the Turkish population, studies on its chemical composition continue. In 2013, another paper was published and reported comparing the chemical profile of aqueous and ethanolic leaves extracts. Results showed higher phenolic contents in ethanolic extract rather than the aqueous one. Flavonoids, chlorogenic acid, gallic and ellagic acid were the most phenolic compounds in *C. laurifolius* ethanol extract [79]. The aerial parts of *C. creticus* from the Island of Crete were analyzed by IR, CID-MS, NMR, DEPT. Nine labdane-type diterpenes were found [53]. In 2012, sonication testing was analyzed by GC/FID, and GC/MS concluded the identification of 24 compounds, also dominated by labdane-type diterpenes [51]. To find a correlation between the activities and biomolecules, a team of Micol was carried out on the composition of different extracts of 4 *Cistus* species abundant in Spanish semi-arid regions. The semiqualitative analysis by RRLC-ESI-TOF-MS of the composition of *Cistus clusii* Dunal, *C. albidus*, *C. ladaniferus*, and *C. salviifolius* did not reveal a significant difference between the four *Cistus* extracts. The presence of phenylpropanoid compounds (phenolic acids, falavonoids, and tannins derivatives) in all species should be mentioned; the proportions were varied. Cyclohexane carboxylic acids, gallic acid, glycosylated quercetin, and myricetin were especially abundant in the *C. clusii*, *C. albidus*, *C. salviifolius* extracts. In contrast, labdanoic acid, betuloside, apigenin, kaempferol, and galloylated flavanols were more abundant in those extracts of *C. ladaniferus* and *C. clusii* [80].

## 3. Biological Potent of *Cistus* Species

All *Cistus* species produce several compounds with pharmacological properties. The international literature has reported the biological activities of *Cistus* species, which are widely known in folk and traditional medicine around the Mediterranean basin.

### 3.1. C. ladaniferus L.

Extracts from *C. ladaniferus* showed to have an antiaggregant effect by inhibiting the thrombin [81]. Also, an antispasmodic action confirms its traditional use for treating intestinal aches [82]. An evaluation of aqueous extracts against glycemic and lipidemic status in diabetic rats showed a significant reduction in blood glucose levels (similar to those obtained with glibenclamide) and the total cholesterol triglycerides in plasma [83]. A reduction of systemic blood pressure in two animal models of hypertension, and then it has antihypertensive properties [84]. Analgesic and anti-inflammatory purposes were also detected in this species [85]. Two papers have focused on antioxidant activity and revealed excellent effectiveness against peroxyl radicals [41,55]. Aqueous extracts of *C. ladaniferus* also have anticarcinogenic compounds, which have cytotoxicity against pancreatic cancer cell lines M220, breast cancer cells MCF7/HER2, and JIMT-1 [86]. The first report about the toxicity of *C. ladaniferus* was published by El Kabbaoui using male and female mice, reported that a dose of 3 and 5 g/kg produced a 10% to 30% mortality rate (acute toxicity test) [87].

### 3.2. C. monspeliensis L.

Considerable research on pharmacological proprieties of *C. monspeliensis* has been done. One of the first examples of cytotoxic activity of extracts and compounds of this species was studied by Dimas and his colleagues. They have demonstrated the inhibition of myricetin isolated from the hexane aerial parts extract against nine human leukemic cell lines. They also showed that acetylation of this compound enhances the growth inhibition, decreasing from IC_50_ 50.1 µM to 38.3 µM. It should be noticed that any paymasters’ modification will modify the results [88]. Demetzos showed that a diterpene isolated from hexane extract from leaves of *C. monspeliensis* does not present any cytotoxic or cytostatic activity against any of the nine cell lines tested [89]. On the other hand, Ben Jemia demonstrated that hexane extract from *C. monspeliensis* leaves was rich with many compounds, especially fatty acids, hydrocarbons, and Vitamin E. The compounds showed significant cytotoxicity against human breast cancer cells with IC_50_ 52.44 mg/mL (better than reference drug IC_50_ 142.36 mg/mL) [41]. Nevertheless, the main limitation of those investigations is the absence of toxicity determination of those extracts. Based on this observation, Vitali studied the acute toxicity using *Artemia salina* L., and cytotoxicity on normal human prostate cells, of lyophilized aqueous extract from aerials parts of *C. monspeliensis*. They observed that the toxicity increase after 24 h with a concentration of 5 mg/mL, but the exposure for 48 h to the extract displayed significant growth inhibition from the concentration of 0.1 mg/mL [29]. Demetzos investigated the in vivo anti-inflammatory activity of the ci-clerodane type Diterpene isolated from hexane extract on the back of hairless mice. The topical application did not seem to have any outcome to the skin barrier repair. Compared to Sayah, who found that aerial parts aqueous extract has a potent effect against carrageenan and analgesic reaction at a dose of 500 mg/Kg body weight [85,89]. This effect may be due to the richness of polyphenolic compounds in the aqueous extract and the lack of epoxide structure in the cis-clerodane molecule. Other researchers reported that methanolic or ethanolic extracts of *C. monspeliensis* are rich in polyphenolic compounds related to antioxidant activity because they can chelate transition metals and prevent them from participating in metal-catalyzed initiation and hydroperoxide decomposition reactions [78,90].

EO’s use as a food supplement for neuroprotective proprieties has also been investigated by M.R. Loizzo, aiming acetylcholinesterase (AChE) butyrylcholinesterase (BChE) enzyme. EO of *C. monspeliensis* does not have any activity against AChE and a slight BChE inhibitory. This observation could be due to the limited amount of compounds that belong to monoterpenes with AChE inhibitory potent such as α-pinene, β-pinene, and α-terpinene. On the other hand, plenty of diterpenes was responsible for the highest lipid peroxidation inhibition [62]. Using in vitro model, Sayah demonstrated the strong effect of aqueous and hydro-methanolic extracts to inhibit α-glucosidase and α-amylase activity (enzymes responsible for the degradation of oligosaccharides into glucose and then increase his level in blood). This inhibition could then be essential in managing blood glucose levels in diabetic patients and preventing type 2 diabetes. The same researchers also established the correlation between total phenolic content, enzyme inhibition, and different antioxidant activities (DPPH, ABTS, FRAP) [91].

### 3.3. C. salviifolius L.

Numerous studies have been conducted to evaluate biologicals proprieties of *C. salviifolius* compounds. Oxidative stress is an essential factor included in chronic and degenerative diseases and is targeted by researchers. Studies on different extracts of *C. salviifolius* showed an interesting source of natural antioxidants. A study was done by Fadi Qa’dan using Fremy’s salt assay to demonstrate the ability of a purified proanthocyanidin from three fractions to reduce free radicals [92]. Additionally, S.K. El Euch aimed to compare methanolic extract from leaves and flower buds, targeting different antioxidant activity tests (DPPH, ABTS, FRAP), enzyme inhibition (xanthine-oxidase XOD, superoxide dismutase SOD, and AChE), anti-inflammatory and cytotoxic activities. In general, the results revealed that extract’s organ origin influenced considerably chemical composition and then biological activities. The highest antioxidant and cytotoxic amounts were observed in flower buds extract, rich with phenolic and flavonoid compounds. On the other hand, leaves extract was rich with tannin compounds and had an inhibitory potent against XOD, SOD, AChE, and anti-inflammatory activity [93]. Another study with aqueous and hydromethanolic aerial parts of Moroccan *C. salviifolius* by K. Sayah also showed a significant phenolic and flavonoid content and effective antioxidant activities. Still, if IC_50_ values of each test were compared, we will find a difference that may be explained by many parameters such as geographical area, organ origin, extraction methods, Etc. Extracts used in this study also exhibited a strong inhibitory toward α-amylase and α-glucosidase, influencing glucose blood levels [91]. To our best knowledge, the survey carried by M.R. Loizzo is the only in literature focused on the antioxidant activity of *C. salviifolius* EO. EO was able to scavenger DPPH and ABTS radicals to reduce antioxidant compounds on ferric tripyridyltriazine (FRAP assay) and inhibit lipid peroxidation (β-carotene), also inhibit AChE and BChE. IC_50_ values reported in this study are much lower compared to those noted with organic extracts. This difference may be attributable to the proportion of different chemical compounds present because EO is a complex mixture of several molecules which can act in synergy or by one or two main components. However, this supports EO’s practical use as a food additive and herbal tea because compounds lipophilic could be delivered by the bloodstream to the brain, an area affected by oxidative damage [62].

Considering the importance of developing pharmaceutical products with lesser side effects for inflammation and pain treatments. The current study proposed by [94] reports the anti-inflammatory and analgesic potent of Moroccan *C. salviifolius* aqueous extracts, an important reduction of inducing paw edema (97.57%), and significant inhibition of writhes for a dose of 500 mg/Kg of body weight. In a recent paper published by I. Chiocchio, the authors demonstrated for the first time the skin protection effect of *C. salviifolius* hydro-methanolic extracts [95]. Trials aimed to test the *in vitro* inhibition of two enzymes which are cosmetic targets (tyrosinase and elastase), showed the inhibition of 51% against elastase and 61% against tyrosinase at a concentration of 50 µg/mL. Also, a correlation was established between inhibition enzymatic potency and the total phenolic and flavonoids content.

### 3.4. C. albidus L.

Ethnopharmacological data revealed the use of *C. albidus* flowering top in decoction against respiratory disorders by the North-West of Morocco population [96]. In this regard, a study was carried to support the traditional pharmacopeia in Morocco. A high value of total phenolic content and total flavonoid content was registered from ethanolic extract (TPC 112.48 ± 1.78 mg GAE/g extract; TFC 24.55 ± 0.58 mg QE/g extract). Those compounds are secondary metabolites that could act as antibacterial responsible for respiratory infections [90].

### 3.5. Cistus heterophyllus subsp. Carthaginensis (Pau)

The influence of environmental conditions in the production of phenolic compounds is a fact. A study carried on how the medium composition affects both the performance and the antioxidant capacity of *C. heterophyllus* shoots cultured in vitro. The finding suggested modulation of parameters suited by physical state and chemical composition. Still, a correlation between phenolic compound levels and the antioxidant capacity (evaluated by DPPH assay) in shoots extracts showed the prominent role of defense against oxidative stress [97].

### 3.6. C. clussii Dunal

In contrast, a study carried out with *C. clussi* on the role of a secondary metabolic pathway in responses to stress such as summer drought, reported the increased syntheses of many phenolic compounds and the maximum efficiency of photosystem II. The induced drought may be due to the compound’s action to protect the plant from oxidative damage [98].

### 3.7. Cistus populifolius L.

The *C. populifolius* aqueous extract from Spain have demonstrated for the first time a high polyphenol (especially ellagitannins) content and high antioxidant capacity observed in many analysis, data reported in this study suggests the use of this species in the food industry and biological systems [86].

### 3.8. Cistus crispus L.

Mainly *C. crispus* was studied by A. Bouyahya’s team. It is used as a poultice applied against wounds [96]. It is well known that secondary metabolites are infused by the solvent used for the extraction, thus affecting biological activities. *C. cripus* methanolic extract showed a higher TPC and TFC, which are the most widely used phenolic substances [99]. Based on our knowledge, there is only one report discussing the ability of *C. crispus* n-hexane extract to act against tumor cells (embryonal rhabdomyosarcoma cancerous) without affecting normal cells (high selectivity indexes) and remarkable IC_50_ value. This finding is interesting, while the absence of correlation between antioxidant and antiproliferative activities supports other targets of phenolic compounds on cancer cell lines [100].

### 3.9. Cistus libanotis L. and Cistus villosus L.

Similar to the previous investigation on the *Cistus* genus, *C. libanotis*, and *C. villosus*, we report the leading presence of flavonoid compounds with other phenolic substances in hexane extract without any antiproliferative potent against three different cell lines [41]. However, the same species’ methanolic extracts also have a significant amount of phenolic, flavonoid, and tannins content. That could prevent the participation of ions in metal-catalyzed initiation and hydroperoxide decomposition reaction. Indeed, this mechanism has been reported to escape oxidative stress, thus prevent many chronic diseases [78]. Moreover, the EO from Italian *C. libanotis* had a fraction composed mainly of Monoterpene hydrocarbons, with the most significant antioxidant potent studied by DPPH, ABTS, FRAP, β-carotene bleaching test, AChE, and BChE. Except for *C. villosus*, which does not have a FRAP activity and low ABTS inhibition [62].

### 3.10. Cistus creticus L.

Investigation on biological activities of *C. creticus* has started since 1994. Many compounds have been isolated from resin and hexane extract, especially labdane type-diterpenes, which exhibited interesting cytostatic and cytotoxic effects against variable human leukemic cell lines with a promising IC_50_ suggesting an induction of apoptosis via p-53i independent pathway [89,97,101]. Further in vivo use is hindered by their water-insolubility. To overcome that, liposome formulation was used to encapsulate two compounds and compare their activity against human cancer cell lines with the free compounds. It has been proven that encapsulation may have several advantages, but it is also important to signal that it may modify the efficacity (not the same IC_50_) [102]. Some of those labdane types were also evaluated for their anti-inflammatory activity in vivo on the back of hairless mice. Still, one of the tested compounds has shown a significant contribution to the skin barrier repair compared to the control, which may be due to the lack of the tested molecule’s epoxide structure [89].

Research on biotechnology to produce secondary metabolites has received particular interest. Marijana has successfully established in vitro culture of the shoots and roots of *C. creticus*, which presented a similarity in secondary metabolites with those found in the native plant, which offers an excellent opportunity for further commercial use while respecting the ecosystem [103].

The south-Moroccan population has reported leaf decoction in the traditional treatment of diabetes [104]. It has also recently been declared that it had a positive influence on the foraging field containing *C. creticus* on the quality of goat milk [105].

Known as *C. incanus*, Italy’s aqueous extract has shown to act as spasmolytic on intestinal and vascular smooth muscle. Its action is concentration-dependently and reversible inhibitory. This finding supports local folk medicine’s beneficial effect on diarrhea and digestive disorders [106]. As known, compounds belonging to labdane-type diterpenes were subject to much research and demonstrated their cytostatic and cytotoxic ability. A study aimed to prepare liposomes and incorporate sclareol has shown a notable reduction in human cancer tumor growth rate than free sclareol [107].

On the other hand, underlining men’s life quality impairs because of benign prostatic hypertrophy. The aqueous extract has been active in suppressing the growth of human epithelial prostate cells. This might be used for treatment since they do not reveal significant toxicity [29]. A polyphenol-rich extract from herb *C. incanus* has increased intracellular oxidative stress in Chinese hamster pulmonary fibroblast cells cultured [108].

Indeed, many mechanisms were suggested, such as regulation-free radical processes as a signal of the transduction pathway or the pro-oxidant activity of polyphenols and their ability to inhibit and reduce the P-gp protein efflux. An evaluation of oxidant/antioxidant status, estrogenic/anti-estrogenic activity, and gene expression profile in mice fed with *Cistus incanus* reach bee pollen, revealed a very noticeable health-protective potential and antioxidant activity [77]. Considering the important use of those antioxidants in the human diet, attention has been paid to herbal infusion, which promoted a high content of phenolic substances and strong antioxidant potent.

### 3.11. Cistus laurifolius L.

*C*. *laurifolius* is mainly known in Turkish folk medicine to treat various types of pain and disorders. As a part of evaluating this traditional use, numerous studies have been conducted. It has been demonstrated in vivo by many modules the anti-inflammatory effect of leaves extracts against multiple molecules, which play a crucial role in inflammatory and immune response such as IL-1, TNF-α, prostaglandins by inducing inflammation in mice. The results were interesting. Extracts and molecules isolated could exert a strong antinociceptive and anti-inflammatory potent without causing any apparent acute toxicity or damage [109,110,111]. The chloroform extract could also exert an analgesic effect by inhibiting tail-flick response rapidly compared with morphine [112]. Another striking point was the in vivo antioxidant activity of extract and compounds isolation, observing possible liver damage and acute toxicity. The potent studied was more than ascorbic acid [113,114]. Whereas the burden of diabetes and its complications increases, specific attention is given to the utilization of tea leaves from *C. laurifolius* to explore its potential effect as antidiabetic. It is a possible influence on platelet aggregation and blood coagulation. Results indicated that this species might also benefit diabetic patients [79,115].

Upon reviewing the literature on the *Cistus* genus, it is found that numerous studies have been carried out on multiple features, uses, and valuables activities which we have mentioned above. To our best knowledge, the first review about the *Cistus* genus has been published by Papaefthimiou. It has summarized an overview of the main *Cistus* species present in the Mediterranean basin [116]. More recently, Stępień also has published a review paper about the biological properties of *Cistus* species [117]. Both teams have highlighted mainly the studies showing promising potential on numerous biological applications without showing value details.

As known multidrug-resistant microorganisms have become increasingly problematic, new drugs are essential to tackle this ever-increasing problem. This document’s quest was to summarize and update recent scientific investigations to determine anti-infective activities separately (antiviral, antiparasitic, antifungal, antibacterial, and antibiofilm). Also classified antibacterial potency on three levels: a—Effect of EO, b—Effect of isolated compounds, and c—Effect of crude extracts.

## 4. Antiviral Activity of *Cistus* Extracts

Viral infections are life-threatening diseases because of their rapid outbreak. Among the most aggressive viral infections are Ebola, AIDS, influenza, and SARS. For instance, influenza is responsible for over 3 million new severe disease cases and 300,000–500,000 deaths yearly [118]. Alarmingly, the number of patients diagnosed with viral infections is increasing every year. For example, the covid-19 pandemic alone infected about 141 M people and caused 3.1 M deaths (WHO 21/04/2021). Treatment of viral diseases is a great challenge even today because of the virus’s adaptation, the emergence of resistant viral pathogens, new viral strains, the high cost and side effects of medicine, and host resistance antiviral drugs [119].

Herbal drugs have gained much importance due to their easy adaptability, low cost, and fewer side reactions on patients [120]. Most studies related to traditional *Cistus* specie’s uses have been carried out in Greece and Turkey.

Three *Cistus* have been reportedly studied for their in vitro and in vivo activity against viral pathogens. Different locations, parts of the plant, and solvents have been used to prepare extracts against H7N7, H1N1, H5N1, HIV, Ebola virus, HSV-1, PI-3, and dengue virus (Table 1).

Dr Pandalis developed a selective extract CYSTUS052 from a distinct variety of *C. incanus*, with a content of more than 26% of polyphenol. This variety is the main ingredient for several products (Cream, tea, syrup, bio pastilles, decoction, salve, food supplements). This extract has demonstrated the greatest antiviral action against influenza A (H7N7) in in vitro (90% inhibition) but also in vivo model (mice treated with aerosols of *Cistus* extract were protected from infection). All experiments concluded on the safety of extracts without toxicity or adverse effects on systemic immune response neither epithelial bronchioles cells, suggesting a direct interaction of polymeric polyphenols with viruses before infection leading to the prevention of absorption in the cells [121]. Ehrhardt et al. attempted to demonstrate anti-influenza activity against other subtype strains (H1N1, H7N7, H5N1). Overall results showed the extract’s ability to reduce the progeny virus titers of different strains with a dose-dependent response [122]. Following safety analysis, the extract did not show any harmful effect on cells. Moreover, confirmation of the previous hypothesis (reduction of the progeny virus titers) was demonstrated using several experiments to indicate that components of the CYSTUS052 extract can directly be interfering with viral HA to block binding to cellular receptors with an unspecific physical mode of antiviral action (using HRV14 as an example). The model proposed overcame resistance problems by mutations of amino-acid residues.

Since the first International Conference on Traditional Medicine and AIDS was held in Dakar in 1999, highlighting the considerable support of medicinal plants among HIV-infected individuals, HIV/AIDS was selected as a priority for future research and development in the area of medicinal plants. The Canadian AIDS Treatment Information Exchange (CATIE) has prepared a list of medicinal plants showing potential beneficial effects for HIV-infected individuals “Decade for the Development of African Traditional Medicine” (2000). In this interest and based on antiviral potent demonstrated by CYSTUS052, Rebensburg showed the inhibitory potent of aqueous extracts and the polyphenol-enriched fraction of *C. incanus* against clinical isolation of HIV with multidrug resistance profile, moreover, the potency of these extracts against emerging viruses such Ebola virus and Marburg virus. The inhibition percentages were more than 80% and an EC50 ranging from 5.40 to 15.06 µg/mL. These results have led to further investigation of the mode of action using different systems (EASY-HIT, TOA, GFP-labelled HIV-1 particles) and the observation demonstrating that *C. incanus* blocks viral particles’ attachment to which inhibit viral entry into host cells. In addition, it blocks gp-120 envelope protein in HIV-1 and envelopes glycoproteins of Ebola and Marburg viruses, which prevent these viral proteins from mediating infection. Even after long-term *C. incanus* treatment (168 days), viruses did not show any resistance. This is presumably attributed to the combination of actives molecules in the extracts compared to treatment with a single-compound antiretroviral drug.

Although these findings were under cell culture conditions and did not identify the individual antiviral agents, it is still believed to have a therapeutic application after extensive investigations, mainly as topical microbicides to prevent STD [123]. Based on the utilization of oleoresin of *C. incanus* as the main ingredient of “Aliptamuscata” by doctors during epidemics of the medieval period for prevention [124] and to ensure a much investigation of *C. incanus* antiviral potency, Kuchta has explored the raw extract and fractions of labdanum resin against the dengue virus, leading to severe forms of the disease. Unfortunately, it had a strong cytotoxic effect. Simultaneously, two subfractions of dichloromethane fraction showed the intense anti-dengue activity of 100% with non-toxic concentration (31.25 µg/mL). The chemical composition revealed epi-manoyloxide, 3-hydroxy-manoyloxide, manoyloxide, 3-acetoxy-manoyloxide [125]. The authors have highlighted an interesting point in this study: why water-soluble polyphenols had the greatest antiviral potency? In contrast, in this case, the water-soluble fraction has enhanced viral proliferation, which may be because the aqueous fraction was previously washed with a solvent much more polar.

An initial finding of antiviral potent of *C. laurifolius* in leaves was reported against HSV-1 and PI-3. No % of inhibition was written. However, the maximum non-toxic concentration measured revealed that hexane fraction had significant activity against PI-3, like the reference drug oseltamivir (32 µg/mL). On the other hand, HSV-1 was less sensitive, with the maximum cytopathogenic effect of 16–32 µg/mL. It is still an interesting finding through the insufficient data since HSV infections are usually severe problems among immunocompromised individuals [126].

**Table 1:** Antiviral potent of *Cistus* extracts.

*Cistus* Species	Collection Area	Part of Plant	Type of Extract	Viruses	Technique	Concentration µg/mL	% of Inhibition	Mechanism	References
*C. incanus* *PANDALIS*	Northern Greece	ND	CYSTUS052 solved in sterile PBS and sterile H_2_O	H7N7	Cell culture and mouse infection model	10,000	90%	Binding with virus and prevent adsorption to cells	[121]
*C. incanus* *PANDALIS*	Northern Greece	ND	CYSTUS052 solved in sterile PBS	H1N1 H7N7 H5N1	Cell culture and plaque titration of progeny virus	50	Maximum reduction in the order of two logs	Interaction of CYSTUS052 components with viral protein surface and block binding to cellular receptors	[122]
*C. incanus* CYSTUS052^®®^ and Cystus^®®^	Northern Greece Germany	ND	Aqueous extract and Polyphenol-enriched fraction	HIV Ebola virus Marburg virus	Cell culture and EASY-HIT technology for identification of HIV inhibitors	100	More than 80%	Blocking the viral gp120-mediated binding of virus protein to heparin and prevent primary attachment to host cells	[123]
*C. laurifolius*	Kurtboğazı, Ankara, Turkey	Leaves	Ethanolic Extract and its fractions	HSV-1 PI-3	Cell culture and Maximum cytopathogenic effect.	512	Not determined most activity with hexane (32 µg/mL MNTC)	ND	[126]
*C. creticus*	Northern Crete	Labdanum resin	Diethyl ether and its fractions	Dengue virus	Cell culture and MTT-test	31.25	100%	Based on literature: neuraminidase inhabiting and blocking viral proteins to prevent infection of host cells	[125]

ND: Not determined; H7N7: Avian influenza virus A/FPV/Bratislava/79; H1N1: Human influenza virus A/Puerto Rico/8/34; H5N1: Human influenza isolate A/Thailand/1(KAN-1)/2004; HIV: Human immunodeficiency virus; HSV-1: Human alphaherpesvirus 1; PI-3:Parainfluenza type 3.

## 5. Antiparasitic Activity of *Cistus* Extracts

Each year diseases caused by parasites lead to hundreds of millions of infected people, particularly in tropical and subtropical regions, resulting in one million deaths [127]. At first report about the potential of *Cistus* against parasites, Fokialakis have demonstrated the significant antileishmanial activity of raw extract of *C. monspeliens* is and *C. creticus* [128,129]. Also, the authors tested pure and semisynthetic compounds from the same species against *L. donovani* promastigote (causative agent of visceral leishmaniasis). Obviously, among the eight natural compounds from *C. monspeliensis*, 18-acetoxy-cis-clerod-3-en-15-ol was the most active with an IC_50_ value of 3.3 µg/mL, while *C. creticus* compounds were less sensitive ent-3b-acetoxy-13-epi-manoyl oxide active with an IC_50_ value of 17 µg/mL. As far as we could observe, semisynthetic derivatives showed variable responses, ranging from inactivated to more active than the parent compound. As it is commonly acknowledged that the fundamental concepts of activity are about selective toxicity against the target without any toxicity to the host cell, none of all compounds tested in this study was cytotoxic to mammalian cells up to the highest concentration of 47.6 µg/mL.

A recent report by Bouyahya on macerated extracts from *C. crispus* leaves against three *Leishmania* species tests at the promastigote stage have demonstrated that among the solvent used, methanol and ethanol, n-hexane showed the best anti-promastigote activity regarding *L. major*, *L. tropica*, and *L. infantum*. Moreover, *L. Infantum* was the most sensitive IC_50_ 47.29 µg/mL. Also, it should not be forgotten that all results obtained with *C. crispus* extracts were lower than the control used (Glucantime^®®^ IC_50_ > 500 µg/mL) [130]. However, no toxicity tests were performed. Unfortunately, there is a high probability that the products that have been classified as active against a particular parasite in vitro are likely to be known toxins to host cells (Table 2).

The in vivo screen should assess activity against an intracellular stage of parasite development to be genuinely representative for an antiparasitic product suitable for development. Nevertheless, no screening system is perfect because of the lack of correlation between tests in vitro and in vivo in all areas of drug discovery. We cannot emulate the complex situation in vivo.

## 6. Antifungal Activity of *Cistus* Extracts

Even though fungi were the first source of antibacterial agents, they are also responsible for fruits and vegetable diseases, like humans affecting the quality of life, causing pain, discomfort, and psychological symptoms. This concern is more severe with the increase of the resistance phenomena.

In search for antifungal drugs in the *Cistus* genus, rockrose is also known as *C. creticus* = *C. villosus* = *C. incanus*, was widely investigated. First, by Demetzos et al., in both reports about EO’s composition and its effect against *C. albicans* which had almost the same effect [69,70]. Comparative MIC analyses of Moroccan and Turkish rockrose against *C. glabrata* and *C. albicans* show respectively significant MIC values with methanol extract from Moroccan species 0.19–6.25 mg/mL and 8–32 mg/mL with aqueous extract from Turkish samples. Interestingly the bio-guided extraction (fractionation with increasing polarity solvent) of Bouamama allowed a better activity localization. Butanol fraction and remaining aqueous layer were more active than the raw extract. It also should be noticed that *C. glabrata* was the most sensitive while *C. krusei* was the most resistant to extracts [31,131]. However, the highest anti-candida activity was reported with phenolic extract of *C. ladaniferus*. MIC was lower 0.05 mg/mL for *C. albicans*, *C. glabrata*, and *C. parapsilosis* [132]. This extract was mainly made of phenolic compounds, which might explain its activity.

Meanwhile, it has been established that EO, aqueous and organic extracts of rockrose are also active against *Aspergillus* species [131,133,134]. Hydro-methanolic extract of *C. incanus* showed a pretty interesting finding against *A. parasiticus* since, at a concentration of 0.2 mg/mL, it inhibited the growth in a percentage of 46% and also inhibited the production of Aflatoxin B1 from 72.5% to 90.1% [30]. It is such an outstanding finding considering Aflatoxin’s risk and damage to crops and to human health, which is supposed to use these extracts as natural products for biological control. Other studies indicate the potential of extracts derived from rockrose in protection against post-harvest diseases. Ameziane reported activity against *P. digitatum*, *P. italium*, *G. candidum* with mycelial growth inhibition ranging from 27% to 79% using methanol extract. However, they did not test it in vivo against fruits. At the same time, more recently, Talbi demonstrated the tremendous antifungal potential against *G. candidum*. The extract has inhibited mycelial growth and germination spores at 100%. It has decreased the incidence and severity of sour rote disease respectively to 40% and 32% compared with a control group without causing any phytotoxic effect [135,136].

Karim did similar investigations to demonstrate the effectiveness of eight *Cistus* varying types of extracts. Obviously, for anti-*G. citri-aurantii*, water was the best extraction solvent, followed by methanol and chloroform. In both studies at a concentration of 5 mg/mL aqueous and methanolic, extracts of *C. creticus*, *C. albidus*, *C. laurifolius*, *C. monspeliensis*, *C. crispus*, *C. salviifolius*, and *C. populifolius* exhibited a total inhibition of arthrospore germination. However, among all extracts, aqueous ones of *C. salviifolius* and *C. monspeliensis* have strongly inhibited the fungi with MIC values low than 0.625 mg/mL.

In comparison, methanolic extracts of the same species had a MIC of 5 mg/mL. Indeed Karim has also uncovered the significant activity of all tested plant extracts to reduce disease incidence and severity [137,138].

To the best of our knowledge, little is shown about post-harvest citrus fungal pathogens. These studies have pointed out that *Cistus* extracts will widen the list of allelopathic plants to *G. citri-aurantii* growth.

A recent Moroccan report about the ethanolic extract from rockrose against *P. expansum* and *P. digitatum*: the research was done using agar dilution, and the results revealed the sensitivity of *P. digitatum* MIC 1 mg/mL while *P. expansum* was more resistant with MIC of more than 10 mg/mL. Also, the same study has evaluated the effect against unusual fungi such as *C. versicolor*, *G. trabeum*, *P. placenta*, and *C. puteana*, which is known to be wood decomposition. Most of them presenting a resistance against ethanolic extract. However, they were more sensitive against EO [134].

According to studies published about EO of *C. ladaniferus*, firstly, Mrabet have highlighted its activity against various fungi, in which *M. racemous*, a polluting fungus, was the most sensitive to the EO. The activity observed may be due to the presence of sesquiterpenes compounds [139]. Years later, Upadhyay has published a detailed report about the potent inhibition of the tested EO against *A. flavus* (MIC 0.6 µL/mL). Its capacity to suppress Aflatoxin B1 production suggests a mode of action based on inhibition of ergosterol biosynthesis (basic sterol of the fungal cell membrane) and enhancement of ions leakage from fungal cells, which leads to fungal growth inhibition. Also, the reduction of methylglyoxal, thus the removal of Aflatoxin B1 biosynthesis. It is also shown that EO was mainly composed of α-asarone, known for its antimicrobial quality but also its toxicity. However, the phytotoxicity investigation has proved EO’s safety for seeds used [140]. Table 3 and Table 4 summarize the work done on the fungi.

## 7. Antibacterial Activity of *Cistus* Extracts

Following the worldwide epidemiology of bacterial infections, antibiotic resistance has become a global pandemic and one of the most considerable worldwide anxieties. The spread of MDR, XDR, and PDR bacteria suggests that in 2050 the leading cause of death will be antibiotic resistance. World Bank has indicated that the number of deaths will rise to 10 million every year if no action is taken.

The development of new molecules of antibiotics is crucial mainly for the treatment of Gram-negative infections such as EPC (Enterobacterales Producing Carbapenemases), ABIR (*Acinetobacter baumannii* Imipenem Resistant), and PAIR (*Pseudomonas aeruginosa* Imipenem Resistant). Also, considering all data about these concerns, to date, several plants have been identified from ethnobotanical information as having valuable properties. Most research into these effects on bacterial infections has been undertaken using EO, aqueous, or organic extracts from purified compounds. Here we analyze the studies about *Cistus* antibacterial impact by type of extract. First EO followed by isolated compounds and then raw extracts.

### 7.1. Antibacterial Effect of EO

Plant EO can be extracted from leaves, fruits, stems, and roots. *Cistus* EO has shown having a broad antimicrobial spectrum, being antifungal and antibacterial, as demonstrated by Demetzos et al. in its three reports with *C. creticus* for the first time. EO from leaves and aerial parts shows a similar profile. Their compounds, mainly diterpenes especially manoyl oxide isomers, inhibit the in vitro growth of *S. aureus*, *S. epidermidis*, and *B. subtilis* even at high dilution (1/2000). Simultaneously, Gram-negative strains represented by *E. coli* and *P. aeruginosa* were more resistant to EO’s action [69,70].

Nevertheless, the oil from *C. creticus* resin Ladano tested against three *Staphylococcus* species shows similar activity with MIC value of 2 mg/mL, which was estimated high according to authors compared to the better action of the isolated compound Ent-3β-hydroxy-13-epi-manoyl oxide MIC = 0.1 mg/mL. The first time this diterpene was reported in the literature, resin EO was most known for its richness with sesquiterpene [142]. These two-chemical structures (Labdane-type diterpenes and sesquiterpenes) might be called phytoalexin. *Citsus* plants produce those compounds in response to infections with fungi, bacteria, viruses, and parasites, explaining their effectiveness against microbial strains on in vitro tests.

Following experimental research for antistaphylococcal activity, Guinoiseau [143] demonstrated the potential of *C. ladaniferus* EO and its efficacy against tested strain, ranging from 0.012 mg/mL for acetate fraction to 0.8 mg/mL for EO in coordination with the inhibition zones. These concentrations were much efficient than those reported with *C. creticus* EO [142]. Authors have suggested and attempted to introduce these extracts at their MIC and measure the reduction in the number of CFU/mL over 7 h. Results show that alcohol fraction led to 99.9% inhibition in less than one hour; furthermore, they act without cell lysis or drastic cell wall damage. Indeed, the low density of plant oils and their rapid diffusion across cell membranes due to their hydrophobicity might support the non-specific inhibition by disturbing ATPase efficiency or the proton motor force, thus blocking the cell division [143]. This propriety might also offer an alternative delivery route, including transcutaneous delivery after scarification or patch application for burns or skin infections. Even though a screening on agar-diffusion, the available evidence of the effectiveness of *C. ladaniferus* EO against Gram-positive strains could not be excluded. It has been reported by Viera [144] that this EO has strongly inhibited a Methicillin-resistant *S. aureus* but also *S. pneumoniae*. Moreover, these activities include inhibition of *E. coli* ESBL producer, which more sensitive (20 mm) than the *E. coli ATCC* 25922 (10 mm). However, a recent study aimed with several EOs *C. ladaniferus* EO inhibited *S. aureus* with a high MIC (0.4 mg/mL). These observations raise several questions of parameters influencing activity, which need further research. The phytochemical data indicate the high content in α-pinene, camphene, fenchone, bornyl acetate, and viridiflorol [143,144]. Despite this, a study carried out the same EO against several strains, including both references and clinical isolates of Gram-negative and positive, did not show any activity except for *L. monocytogenes* whose diameter of zone inhibition was 19 mm [145]. This may eventually be due to the low volume deposited per disc (3 µL) (Table 5).

### 7.2. Antibacterial Effect of Isolated Compounds

However, little work has been carried out using purified compounds. Several isolated compounds from *Cistus* species have definite activity in laboratory trials. In addition to *C. creticus*, in which Demetzos [142] have provided action against *Staphylococcus*, the team’s investigation continued to isolate other compounds that have antibacterial activity. Years later, they report a novel clerodane diterpene called (+)-19-acetoxy-cis-clerodan-3-ene-15-oic acid from *C. monspeliensis* leaves extract tested toward Gram-positive and Gram-negative models. The results showed that the smaller concentration of 0.031 mg/mL used induced inhibition of all *Staphylococcus* growths at this concentration, no inhibition of Gram-negative. When the concentration increases, the compound had a MIC of 0.499 mg/mL against *E. coli*, *K. pneumoniae*, *P. aeruginosa* [147]. Recently, a work published by Móricz [148] was undertaken to investigate and identify antibacterial components from several *C. incanus* herbal tea. Experiments focused on multi-step extraction to select a fraction that contains flavonoid aglycone.

Additionally, the antibacterial profile of reported components (Apigenin, Kaempferide, Cis-tiliroside/trans-tiliroside, p-coumaric acid-conjugated tiliroside) against *B. subtilis* and *A. fischeri*. The determination was done using a simple, rapid, and inexpensive method, namely thin-layer chromatography-direct bioautography (TLC-DB). The bioautograms discovered were corresponding to the total inhibition of both strains. Furthermore, this study has shown the ineffectiveness of the raw extract in comparison to the isolated faction. Indeed, the authors have highlighted the side effect of the usual fractionation protocols whose formation of artifacts may be present. It is generally well known that artifacts lead to loss of activity, the formation of active compounds from inactivating ones, Right away toxic compounds, and the difficulty of reproducing an extraction or purification method. These products and their published activity are recorded in Table 6.

### 7.3. Antibacterial Effect of Crude Extracts

Nevertheless, raw extracts and liquid-liquid fractions were widely used to explore the antibacterial potential. *S. aureus* and *E. coli* were the most studied strains in all cases. Among the *Cistus* extracts investigated, ethanolic and its fractions from *C. laurifolius* exhibited the best inhibition activity against *S. aureus* (MIC = 0.064 mg/mL) and *E. coli* (MIC = 0.032 mg/mL) [126] in comparison with other species [31,86,99]. A quiet observation about the activity of *C. monspeliensis* in two studies revealed a difference in MIC while it was about the same strain and same solvent (methanol). Bouamama [131] reports a MIC value of 25 mg/mL while Ben Sassi [149] have found 0.312 mg/mL. However, none of the extracts in this study could inhibit *E. coli*, reinforcing the suggestion about a difference in regions’ chemotype.

Suppose carrying out a comparison of other species from each Gram class to attempt an extrapolation of activity observed with *E. coli* and *S. aureus*, and considering the risk in which such species *K. pneumoniae*, *P. aeruginosa*, *A. baumannii*, *E. hireae*, and *L. monocytogenes* may be incriminated (food safety issues, nosocomially acquired infections...). Data analysis of the subsequent studies [86,143,149,150,151] showed that we could not cluster inhibition activity following bacterial species or *Cistus* species. The same extract might exert a different effect on strains from the same Gram class, even from the same genus. For example, raw extract of *C. monspeliensis* demonstrated a significant inhibition against *P. aeruginosa* (MIC = 0.625 mg/mL) while it did not affect *K. pneumoniae* [149], which was sensitive to ethanolic extract from *C. laurifolius* (MIC = 0.032 mg/mL) [126]. However, if taken independently*, L. monocytogenes* was more sensitive to ethanolic extract from *C. salviifolius* (MIC = 0.515 mg/mL) [152] than ethanolic extract from *C. crispus* (MIC = 8 mg/mL) [99]. Based on these observations and the chemical class differences in each solvent used for extraction, the report of Ustun [126] raises several questions about how a strain presents the same sensitivity profile against all extracts tested (MIC of *A. baumannii* 0.032 with hexane, chloroform, butanol, ethanol and remaining aqueous extracts).

On the other hand, few studies were done with clinical isolates and resistant strains. According to Ben Sassi [149], none of the tested extracts was active against isolated bacteria (*E. coli*, *K. penumoniae*, *E. cloaceae*, *S. marcescens*, *A. hyrdophila*, *S. aureus*) except for *S. epidermidis and S. saprophyticus*. Viapiana [31] have reported the activity of aqueous extract of *C. incanus* against MRSE (MIC 1 mg/mL) and MRSA (MIC 0.5 mg/mL). Furthermore, this is the only work done against *H. pylori* and *S. pyogenes*. This might be due to difficulty maintaining cultures. However, MICs reports were moderate (64 and 8 mg/mL for *H. pylori* and S*. pyogenes*, respectively). Nevertheless, there is great hope with this species since *C. incanus* is widely used as herbal tea.

A short communication published in Natural Product Research reports the potential of ethanolic extract of *C. ladaniferus* against several strains. Clinical samples of *H. pylori* were more sensitive than reference strain. Also *B. cereus* was inhibited at 0.625 mg/mL [153]. Ahlem [154] and Mahmoudi have demonstrated the overall activity of leaves ethanolic extract from *C. monspeliensis* compared to *C. salviifolius*. However, the patterns of inhibition varied.

It is not entirely clear why phytochemists and microbiologists have not further researched mycobacteria, even if WHO reports a total of 1.5 million people died from tuberculosis in 2018. Pharmaceutical companies are spending many resources searching for new agents. To our best knowledge, the only report about the antimycobacterial effect with *Cistus* extracts is from *C. albidus*, *C. salviifolius*, and *C. monspeliensis* by the Iraqui team. Results show complete inhibition of these species against atypical mycobacteria and *M. bovis*. Even the lack of details and referenced methods to study the activity against this particular strain gives us hope that further research may eventually lead to potential agents [155,156].

The very nature of the disease being studied may play a significant role in whether effective natural products will be identified for its treatment. Interestingly, patients reported that the ingestion of tea infusion and aqueous dry extract in the capsule from *C. creticus* had improved their clinical conditions related to Lyme borreliosis disease. Hence, it was confirmed first by Hutschenreuther [68] whose total stagnation of *B. burgdorferi s.s* in vitro growth at 0.2% concentration of EO. EO was dominated by labdane-type manoyloxides and carvacrol, while none of the polar extracts has exhibited activity against tested strain. Years later, Rauwald [157] aimed to clarify the anti-Borrelia active component already reported. Bioassay-guided procedure on Crete *C. creticus* led to the confirmation and isolation of carvacrol and four manoyloxide, in which epi-manoyloxide was the strongest antispirochaetal whose effect was equal to amoxicillin (Table 7).

A minimal number of studies carried out the anti-adhesive activity of *Cistus* extracts (Table 8). To reinforce the use of *C. incanus* herbal tea (from Dr. Pandalis) as an appropriate alternative available in everyday life and verify its possible exploitation in dentistry as well. The study of Hanning [158] showed that the impact of *cistus*-tea on initial oral biofilm using a model. Indeed, the fluorescence microscopy showed a pronounced reduction of initial bacterial colonization due to rinses with *cistus*-tea. Furthermore, another report in the same field has demonstrated the potential of ethanolic extract from *C. ladaniferus* agains*t P. aeruginosa* marine bacterium, which accelerates the corrosion of 304 L Stainless Steel. The HPLC-Q-TFO-MS analysis revealed many phenolics compounds, which show antibacterial effects and corrosion inhibition [159].

## 8. Conclusions

*Cistus* is a Mediterranean native genus of shrubs belonging to the family *Cistaceae*. Species of this genus can grow during hot summers and after wildfires. *Cistus* species are most widespread in the Mediterranean region, whilst some of them are endemic. *Cistus* plants can grow under slightly different environmental conditions. Most species are very fragrant and sweet-smelling. Studies have reported that *Cistus* species are riches in polyphenolic compounds. Meanwhile, *C. incanus* and *C. albidus* are riche in flavonoids. *C. ladaniferus*, *C. salviifolius*, *C. laurifolius*, *C. monspeliensis*, and *C. libanotis* are mainly enriched ellagitannins and flavonoids. Terpenes were identified in *C. ladaniferus* and *C. laurifolius*. Diterpenes were reported in *C. monspeliensis*, *C. libanotis*, *C. villosus*, and *C. creticus*. While sesquiterpenes were detected in *C. albidus* and *C. salviifolius*.

*Cistus* species have long been used as remedies in traditional folk medicines of different populations around the world. The crude extracts and EO of these herbs have been effective against inflammation, would and skin disease, various microbial infections, diabetes, pain, arterial hypertension. *Cistus* EO is approved by the Food & Drug Administration (FDA) as a food additive and flavoring agent. Due to the increasing interest in natural products and the benefits of *Cistus* species, EO, labdanum, bee pollen, and tea are owning a significant mark in herbal products. It was reported that *C. monspeliensis* has the function of promoting energy metabolism pathways in human intestinal epithelial cells. Extracts from *C. incanus* are used as a dietary supplement to prevent chronic diseases due to their higher and diverse phenolic compounds. These characteristics can be much appreciable for being exploited in the food industry.

Previous summarized works in this review have indicated and confirmed the broad biological potentials of *Cistus* plant extracts. In *in vivo* model, *Cistus* showed anti-influenza, analgesic, anti-inflammatory, and spasmolytic potentials. In the in vitro model, *Cistus* showed promising effects against HIV, Ebola, Marburg virus, and clinical bacteria.

We can confirm that *Cistus* can constitute a great source of drug discovery. However, besides all the beneficial attributes of this plant as well investigated above, it is essential to highlight some limitations. Some species belonging to *Cistus* can exhibit in vivo toxic effects. Moreover, extracts from some *Cistus* species were reported to be active in vitro only.

The present review sheds light on the antimicrobial activity of *Cistus* sp. In this sense, we can conclude that this genus has great potential to serve society as it provides promising chemicals to fight such infections.

## Figures and Tables

**Table 2:** Antiparasitic potent of *Cistus* extracts.

*Cistus* Species	Collection Area	Part of Plant	Type of Extract	Parasite	Technique	Concentration µg/mL	IC_50_ µg/mL	Mechanism	References
*C. monspeliensis* *C. creticus*	Greece	Aerial parts Resin	Pure compounds from Dichloromethane extracts and semisynthetic derivatives	*L. donovani*	In vitro culture of promastigote and Alamar blue assay	1.6–8–40	From 3.5 to 37	ND	[129]
*C. crispus*	Ouezzane, Morocco	Leaves	Methanolic, Ethanolic and *n*-hexane extracts	*L. major* *L. tropica* *L. infantum*	MTT assay	ND	*n*-hexane against *L. major* = 47.29	ND	[130]

ND: Not determined; *L*: *Leishmania*.

**Table 3:** Antifungal potent of *Cistus* extracts.

*Cistus* Species	Collection Area	Part of Plant	Type of Extract	Fungi	MIC µg/mL	MGI %	References
*C. creticus*	Greece	Leaves	EO	*C. albicans*	MID (1/250)	ND	[69]
*C. criticus* subsp. *eriocephalus*	Kandanos Chania, Greece	Aerial parts	EO	*C. albicans*	MID (<1/200)	ND	[70]
*C. criticus* subsp. *creticus*					MID (<1/250)		
*C. ladaniferus*	ND	ND	Essential Oil; Concrete; Absolute and Resinoid	*A. niger* *B. cinerea* *M. racemosus* *V. albo-atrum*	4000 to 10,000 ppm of EO	99.4% (*M. racemosus*)	[139]
*C. villosus*	Ourika, Morocco	Leaves	Methanolic extracts and their fractions	*A. fumigatus* *C. albicans* *C. krusei* *C. glabrata*	100,000 to 200,000 1560 to 200,000 6250 to >200,000 190 to 390	ND	[131]
*C. monspeliensis*				*A. fumigatus* *C. albicans* *C. krusei* *C. glabrata*	25,000 to 200,000 25,000 to 200,000 12,500 to 100,000 1560 to 3125		
*C. villosus*	Agadir, Morocco	Leaves+stem	Plant powders	*P. digitatum* *P. italicum* *G. candidum*	ND	100% 100% 100%	[135]
			EO			37% 30% 18%	
			Methanolic			77% 17% 79%	
			Chloroformic			27% 27% 21%	
*C. villosus*	Agadir, Morocco	Leaves and stem	Aqueous	*G. candidum*	156	100%	[136]
*C. ladanifer*	Montesinho, Portugal	Leaves	Phenolic extract	*C. albicans* *C. tropicalis* *C. glabrata* *C. parapsilosis*	<50 625 <50 <50	ND	[132]
*C. incanus*	Greece	ND	Hydro-methanolic	*A. parasiticus* *A. carbonarius*	ND	45.91% 30.65%	[133]
*C. incanus*	Turkey	Unknown	Aqueous	*C. albicans* *C. glabrata*	32,000 8000	ND	[31]
*C. albidus*	Morocco	Aerial parts	Aqueous	*Geotrichumcitri-aurantii*	>10,000	>80%	[137]
*C. creticus*		Aerial parts			1250	100%	
*C. laurifolius*		Aerial parts			<625	100%	
*C. salviifolius*		Aerial parts			<625	ND	
*C. monspeliensis*		Aerial parts			<625	100%	
*C. ladanifer*		Aerial parts			<625	100%	
*C. crispus*		Aerial parts			>10,000	100%	
*C. populifolius*		Leaves			<625	100%	
*C. creticus*	Tafraout, Morocco	Leaves	EO	*C. versicolor* *G. trabeum* *C. puteana* *P. placenta* *P. digitatum* *P. expansum* *A. niger*	1/64 1/250 1/1000 1/2000 1/100 1/32 1/100	ND	[134]
			Ethanolic		>10,000 1000 >10,000 1000 1000 >10,000 >10,000		
*C. salviifolius* *C. monspeliensis*	Jendouba, Tunisia	Leaves	Ethanol Hexane Water	*C. albicans* *A. niger*	ND	6300–12,500 3100–12,500	[141]

ND: Not determined; *C. albicans*: *Candida albicans*; *A. niger*: *Aspergillus niger*; *B. cinerea*: *Botrytis cinerea*; *M. racemosus*: *Mucor racemosus*: *Verticilliumalbo-atrum*; *A. fumigatus*: *Aspergillus fumigatus*; *C. krusei*: *Candida krusei*; *C. glabrata*: *Candida glabrata*; *P. digitatum*: *Penicillium digitatum*; *P. italicum*: *Penicillium italicum*; *G. candidum*: *Geotrichum candidum*; *C. tropicalis*: *Candida tropicalis*; *C. parapsilosis*: *Candida parapsilosis*; *A. parasiticus*: *Aspergillus parasiticus*; *A. carbonarius*: *Aspergillus carbonarius*; *C. versicolor*: *Coriolus versicolor*; *G. trabeum*: *Gloeophyllum trabeum*; *C. puteana*: *Coniophora puteana*; *P. placenta*: *Poria placenta*; *P. digitatum*: *Penicillium digitatum*; *P. expansum*: *Penicillium expansum*.

**Table 4:** Antifungal and antiaflatoxigenic potent of *Cistus* extracts.

*Cistus* Species	Collection Area	Part of Plant	Type of Extract	Fungi	Fungi Secondary Metabolites	MIC µL/mL	Finding	Mode of Action	References
*C. incanus*	North Greece	ND	Hydro-methanolic	*Aspergillus parasiticus*	Aflatoxin B1	ND	Both mediums (macadamia, YES) inoculated in the presence of *C. incanus* extract showed potent inhibition of AFB1 production ranging from 72.5% to 90.1%	ND	[30]
*C. ladanifer*	ND	ND	Essential Oil	*Aspergillus flavus* AF-M-K5	Aflatoxin B1	0.6	The EO was found to have an intense antifungal activity, with fungistatic action (MFC 3.0 µL/mL). Moreover, it caused absolute suppression of AFB1 at 0.5 µL/mL, without any phytotoxicity against tested seeds	Inhibition of ergosterol biosynthesis and enhancement of ions leakage from fungal cells leads to fungal growth inhibition -Antiaflatoxigenic potential can be attributed to the reduction of methylglyoxal which represses the expression of *aflR*	[140]

**Table 5:** Antibacterial potential of the essential oils of *Cistus.*

*Cistus* Species	Collection Area	Part of Plant	Type of Extract	Tested Bacteria	DZI (mm)	MID	References
*C. creticus*	Greece	Leaves	EO	*S. aureus* *S. epidermidis* *E. coli* *P. aeruginosa* *B. subtilis* *M. luteus*	ND	1/2000 1/500 <1/125 <1/400 1/2000 1/500	[69]
*C. creticus*	Kandanos, Chania, Greece	Aerial parts	EO	*B. subtilis* *S. aureus* *S. epidermidis* *P. aeruginosa* *E. coli*	ND	<1/2000 1/2000 1/500 <1/400 <1/125	[70]
*C. creticus*	Greece	Resin Ladano	EO	*S. aureus* *S. epidermidis* *S. hominis*	ND	2000 µg/mL	[142]
*C. ladaniferus*	Corsica, France	Aerial parts	EO	*S. aureus*	52	800 µg/mL	[143]
			Hydrocarbonated fraction		6.70	50,000 µg/mL	
			Oxygenated fraction		35	100 µg/mL	
			Acetate fraction		30	12.5 µg/mL	
			Alcohol fraction		50	1500 µg/mL	
*C. ladaniferus*	Alentejo, Portugal	Leaves	EO	*S. aureus* *B. subtilis* *S. pneumoniae* *E. coli* *P. aeruginosa* *MRSA* *E. coli ESBL*	11 11 38 10 9 23 20	ND	[144]
*C. ladaniferus*	Unknown	Leaves and branches	EO	*S. aureus*	ND	400	[146]

MID: Maximum Inhibitory Dilution; ND: Not determined; *S. aureus*: *Staphylococcus aureus*; *S. epidermidis*: *Staphylococcus epidermidis*; *E. coli*: *Escherichia coli*; *P. aeruginosa*: *Pseudomonas aeruginosa*; *B. subtilis*: *Bacillus subtilis*; *M. luteus*: *Micrococcusluteus*; *S. hominis*: *Staphylococcus hominis*; *S. pneumonia*: *Streptococcus pneumonia*; *MRSA*: *Methicillin-resistant Staphylococcus aureus*; *E. coli ESBL*: *Escherichia coli Extended Spectrum β-lactamases*.

**Table 6:** Antibacterial potent of isolated compounds of *Cistus.*

Cistus Species	Collection Area	Part of Plant	Isolated Compounds	Tested Bacteria	MIC (µg/mL)	References
*C. creticus*	Greece	Resin Ladano	*Ent*-3*β*-hydroxy-13-*epi*-manoyl oxide	*S. aureus* *S. epidermidis* *S. hominis*	100 100 100	[142]
*C. monspeliensis*	ND	Leaves	*(+)-19-acetoxy-cis-clerodan-3-ene-15-oic acid*	*S. aureus* *S. epidermidis* *S. hominis* *K. pneumoniae* *E. coli* *P. aeruginosa*	31 31 31 499.4 499.4 499.4	[147]
C. incanus *	Turkey	Aerial parts	Apigenin Kaempferide *Cis*-tiliroside*/trans*-tiliroside *p*-coumaric acid-conjugated tiliroside	*B. subtilis* *A. fischeri*	ND	[148]

ND: Not determined; MIC: Minimum Inhibitory Concentration; *S. aureus*: *Staphylococcus aureus*; *S. epidermidis*: *Staphylococcus epidermidis*; *S. hominis*: *Staphylococcus hominis*; *K. pneumoniae*: *Klebsiellapneumoniae*; *E. coli*: *Escherichia coli*; *P. aeruginosa*: *Pseudomonas aeruginosa*; *B. subtilis*: *Bacillus subtilis*; *A. fischeri*: *Aliivibrio fischeri*; *: The antibacterial assay in this study was performed using Thin-layer chromatography-direct bioautography.

**Table 7:** Antibacterial potent of crude extracts of *Cistus.*

*Cistus* Species	Collection Area	Part of Plant	Solvent Used	Sensitive Bacteria	DZI (mm)	MIC (µg/mL)	References
*C. creticus* *C. laurifolius* *C. monspeliensis* *C. parviflorus* L.*C. salviifolius*	Aegean Mediterranean and Inner Anatolian, Turkey	Leavesand fruits	Water Methanol Chloroform Ethyl acetate *n*-butanol	*S. aureus* *B. subtilis* *B. cereus*	9–15 8–10 8–12	ND	[150]
*C. villosus*	Ourika, Marrakesh, Morocco	Leaves	Methanol Hexane Dichloromethane Ethylacetate Butanol	*E. coli* *P. aeruginosa* *S. aureus* *E. hirea*	ND	1560–3125 3125 0.78–1.56 1560–6250	[131]
*C. monspeliensis*						3125–25,000 1560–50,000 1560–25,000 1560–25,000	
*C. albidus* *C. monspeliensis* *C. salviifolius*	Chefchaouen, Morocco	Leaves	Water Ethanol	*M. aurum A^+^* *M. smegmatis MC^2^* *M. bovis* *M. vaccae*	Total inhibition at 160 mg/mL of extracts incorporated in the medium		[155]
*C. monspeliensis*	Tunisia	Leaves and Flowers	Hexane Acetone Methanol	*S. aureus* *S. epidermidis* *S. saprophiticus* *E. faecalis* *P. aeruginosa*	12–23 12–32 10–24 10–18 12–18	156–625 78–1250 312–1250 625–1250 625–1250	[149]
*C. ladaniferus*	Spain	Aerial parts	Water	*S. aureus* *E. coli*	ND	MIC_50_ 154 900	[86]
*C. populifolius*						344 123	
*C. salviifolius*	Unknown	Leaves	Ethanol	*L. monocytogenes*	20	515	[152]
*C. ladaniferus*	Spain	Unknown	Water Hydroalcohol	*S. aureus* *E. coli*	ND	MIC_50_ 144–569 113–612	[151]
*C. albidus*						60–292 233–336	
*C. salviifolius*						45–52 221–289	
*C. clusii*						91–392 116–1064	
*C. monspeliensis*	Ouezzane, Morocco	Leaves	Ethanol	*E. coli K12* *S. aureus*	9 16	ND	[90]
*C. albidus*		Aerial parts			9 17		
*C. crispus*	Ouezzane, Morocco	Leaves	Ethanol *n*-butanol Methanol– Ethyl acetate (fraction)	*E. coli K12*	<8–14	>8000	[99]
				*S. aureus*	15–29	1000–3000	
				*L. monocytogenes*	21–31	1000–8000	
				*P. aeruginosa*	<8–13	1000–>8000	
*C. incanus*	Turkey	Unknown	Water	*S. aureus* *MRSA* *S. epidermidis* *MRSE* *S. pyogenes β* *E. hirae* *B. subtilis* *E. coli* *P. aeruginosa* *H. pylori*	ND	500 500 1000 1000 8000 16,000 8000 8000 8000 64,000	[31]
*C. laurifolius*	Ankara, Turkey	Leaves	Ethanol *n*-hexane Dichloromethane Butanol	*E.coli* *P. aeruginosa* *P. mirabilis* *K. pneumonia* *A. baumannii* *S. aureus* *E. faecalis*	ND	32 64 32 32 32 64 64	[126]
*C. monspeliensis*		Leaves	Methanol	*E. coli* *E. aerogenes* *K. pneumoniae* *P. stuartii* *P. aeruginosa*	13 11 8 10 9	64 68 71 75 65	[160]
*C. ladaniferus*	Taza, Morocco	Leaves	Ethanol	*P. aeruginosa*	ND	2500	[159]
*C. salviifolius*	Sidi Mechreg, Tunisia	Leavesand Flowers	Ethanol	*E. coli* *P. aeruginosa* *S. typhimurium* *S. aureus* *B. subtilis* *L. monocytogenes*	22–24 23–26 21–25 22–25 23–24 20–25	12,500 12,500 12,500 1562–3125 12,500 1562–12,500	[154]
*C. monspeliensis*					11–22 15–25 15–24 15–25 15–24 15–22	12,500 12,500 12,500 1562 12,500 1562–3125	
*C. salviifolius*	Jendouba, Tunisia	Leaves	Ethanol Hexane Water	*E. coli* *S. typhimirium* *P. aeruginosa* *S. aureus* *E. faecalis*	18 20 10 17 13	12,500 25,000 3100 12,500 6300	[141]
*C. monspeliensis*					14 17 10 17 16	6300 12,500 6300 12,500 12,500	

ND: Not determined; DZI: Diameter of Zone Inhibition; MIC: Minimum Inhibitory Concentration; *S. aureus*: *Staphylococcus aureus*; *B. subtilis*: *Bacillus subtilis*; *B. cereus*: *Bacillus cereus*; *E. coli*: *Escherichia coli*; *P. aeruginosa*: *Pseudomonas aeruginosa*; *E. hirea*: *Enterococcus hirea*; *M. aurum A^+^*: *Mycobacterium aurum A^+^*; *M. smegmatis MC^2^*: *Mycobacterium smegmatis MC^2^*; *M. bovis*: *Mycobacterium bovis*; *M. vaccae*: *Mycobacterium vaccae*; *S. epidermidis*: *Staphylococcus epidermidis*; *S. saprophyticus*: *Staphylococcus saprophyticus*; *E. faecalis*: *Enterococcus faecalis*; *L. monocytogenes*: *Listeria monocytogenes*; *MRSA*: *Methicillin-resistant Staphylococcus aureus*; *MRSE*: *Methicillin-resistant Staphylococcus epidermidis*; *S. pyogenes β*: *Streptococcus pyogenes*; *E. hirae*: *Enterococcus hirae*; *H. pylori*: *Helicobacter pylori*; *P. mirabilis*: *Proteus mirabilis*; *K. pneumoniae*: *Klebsiella pneumoniae*; *A. baumannii*: *Acinetobacter baumannii*; *E. aerogenes*: *Enterobacter aerogenes*; *P. stuartii*: *Providencia stuartii*; *S. typhimirium*: *Salmonella typhimirium*.

**Table 8:** Antibiofilm potent of *Cistus* extract.

*Cistus* Species	Collection Area	Part of Plant	Solvent Used	Bacteria Used	Surface Used	Finding	References
*C. incanus* Dr. Pandalis	Northern Greece	Unknown	Water	Oral bacteria	Cylindrical enamel slabs	Rinses with *cistus-*tea rich with polyphenols have reduced initial bacterial colonization (visualized by microscopic fluorescence method) on enamel in situ. Also, enzymes present in the pellicle were conserved and not affected by compounds present in the extract.	[158]
*C. ladaniferus*	Taza, Morocco	Leaves	Ethanol	*P. aeruginosa*	304 L SS coupons	*P. aeruginosa* accelerates the corrosion rate, while the compounds present in ethanolic extract demonstrate a dual effect: damage in the morphology of bacterial cell membrane and adsorption on a surface. That changes physicochemical proprieties and led to the formation of the non-conducting protective layer, which inhibited the 304 L SS coupon surface’s biocorrosion.	[159]

*P. aeruginosa*: *Pseudomonas aeruginosa*.

## Data Availability

The data used to support the findings of this study are available from the corresponding author upon request.

## References

[1] Ekor M. (2014). The growing use of herbal medicines: Issues relating to adverse reactions and challenges in monitoring safety. Front. Pharmacol..

[2] Adeniyi A., Asase A., Ekpe P.K., Asitoakor B.K., Adu-Gyamfi A., Avekor P.Y. (2018). Ethnobotanical study of medicinal plants from Ghana; Confirmation of ethnobotanical uses, and review of biological and toxicological studies on medicinal plants used in Apra Hills Sacred Grove. J. Herb. Med..

[3] Buragohain J. (2011). Ethnomedicinal Plants Used by the ethnic Communities of Tinsukia District of Assam, India. Rec. Res. Sci. Technol..

[4] Mikawlrawng K., Rani R., Kumar S., Bhardwaj A.R., Prakash G. (2018). Anti-paralytic medicinal plants—Review. J. Tradit. Complement. Med..

[5] Cowan M.M. (1999). Plant Products as Antimicrobial Agents. Clin. Microbiol. Rev..

[6] Borris R.P. (1996). Natural products research: Perspectives from a major pharmaceutical company. J. Ethnopharmacol..

[7] Nair J.J., Wilhelm A., Bonnet S.L., van Staden J. (2017). Antibacterial constituents of the plant family *Amaryllidaceae*. Bioorg. Med. Chem. Lett..

[8] Newman D.J., Cragg G.M. (2012). Natural Products As Sources of New Drugs over the 30 Years from 1981 to 2010. J. Nat. Prod..

[9] Cos P., Vlietinck A.J., Berghe D.V., Maes L. (2006). Anti-infective potential of natural products: How to develop a stronger in vitro ‘proof-of-concept’. J. Ethnopharmacol..

[10] Haruna A., Yahaya S.M. (2021). Recent Advances in the Chemistry of Bioactive Compounds from Plants and Soil Microbes: A Review. Chem. Afr..

[11] Asif M., Yehya A.H.S., Al-Mansoub M.A., Revadigar V., Ezzat M.O., Ahamed M.B.K., Oon C.E., Murugaiyah V., Majid A.S.A., Majid A.M.S.A. (2016). Anticancer attributes of *Illicium verum* essential oils against colon cancer. S. Afr. J. Bot..

[12] Liu R.H. (2013). Health-Promoting Components of Fruits and Vegetables in the Diet. Adv. Nutr..

[13] Altemimi A., Lakhssassi N., Baharlouei A., Watson D.G., Lightfoot D.A. (2017). Phytochemicals: Extraction, Isolation, and Identification of Bioactive Compounds from Plant Extracts. Plants.

[14] McClements D.J. (2020). Advances in nanoparticle and microparticle delivery systems for increasing the dispersibility, stability, and bioactivity of phytochemicals. Biotechnol. Adv..

[15] Zhishen J., Mengcheng T., Jianming W. (1999). The determination of flavonoid contents in mulberry and their scavenging effects on superoxide radicals. Food Chem..

[16] Kumar S., Pandey A.K. (2013). Chemistry and Biological Activities of Flavonoids: An Overview. Sci. World J..

[17] Pratt D.E., Watts B.M. (1964). The Antioxidant Activity of Vegetable Extracts I. Flavone Aglycones. J. Food Sci..

[18] Ahmed S.I., Hayat M.Q., Tahir M., Mansoor Q., Ismail M., Keck K., Bates R.B. (2016). Pharmacologically active flavonoids from the anticancer, antioxidant and antimicrobial extracts of *Cassia angustifolia* Vahl. BMC Complement. Altern. Med..

[19] Traka M.H., Mithen R.F. (2011). Plant Science and Human Nutrition: Challenges in Assessing Health-Promoting Properties of Phytochemicals. Plant Cell.

[20] Valenzuela-Grijalva N.V., Pinelli-Saavedra A., Muhlia-Almazan A., Domínguez-Díaz D., González-Ríos H. (2017). Dietary inclusion effects of phytochemicals as growth promoters in animal production. J. Anim. Sci. Technol..

[21] Vijayakumar S., Prabhu S., Rajalakhsmi S., Manogar P. (2016). Review on potential phytocompounds in drug development for Parkinson disease: A pharmacoinformatic approach. Inform. Med. Unlocked.

[22] Parvez S., Kang M., Chung H.-S., Bae H. (2007). Naturally occurring tyrosinase inhibitors: Mechanism and applications in skin health, cosmetics and agriculture industries. Phytother. Res..

[23] Perrino E.V., Tomaselli V., Costa R., Pavone P. (2013). Conservation status of habitats (Directive 92/43 EEC) of coastal and low hill belts in a Mediterranean biodiversity hot spot (Gargano—Italy). Plant Biosyst. Int. J. Deal. Asp. Plant Biol..

[24] Wagensommer R.P., Medagli P., Turco A., Perrino E.V. (2020). IUCN Red List evaluation of the *Orchidaceae* endemic to Apulia (Italy) and considerations on the application of the IUCN protocol to rare species. Nat. Conserv. Res..

[25] Perrino E., Valerio F., Gannouchi A., Trani A., Mezzapesa G. (2021). Ecological and Plant Community Implication on Essential Oils Composition in Useful Wild Officinal Species: A Pilot Case Study in Apulia (Italy). Plants.

[26] Maruca G., Spampinato G., Turiano D., Laghetti G., Musarella C.M. (2019). Ethnobotanical notes about medicinal and useful plants of the Reventino Massif tradition (Calabria region, Southern Italy). Genet. Resour. Crop. Evol..

[27] Singh B., Singh B., Kishor A., Singh S., Bhat M., Surmal O., Musarella C. (2020). Exploring Plant-Based Ethnomedicine and Quantitative Ethnopharmacology: Medicinal Plants Utilized by the Population of Jasrota Hill in Western Himalaya. Sustainability.

[28] Fernández-Mazuecos M., Vargas P. (2010). Ecological rather than geographical isolation dominates Quaternary formation of Mediterranean *Cistus* species. Mol. Ecol..

[29] Vitali F., Pennisi G., Attaguile G., Savoca F., Tita B. (2011). Antiproliferative and cytotoxic activity of extracts from *Cistus incanus* L. And *Cistus monspeliensis* L. on human prostate cell lines. Nat. Prod. Res..

[30] Kalli V., Kollia E., Roidaki A., Proestos C., Markaki P. (2018). *Cistus incanus* L. extract inhibits Aflatoxin B1 production by *Aspergillus parasiticus* in macadamia nuts. Ind. Crop. Prod..

[31] Viapiana A., Konopacka A., Waleron K., Wesolowski M. (2017). *Cistus incanus* L. commercial products as a good source of polyphenols in human diet. Ind. Crop. Prod..

[32] Ellul P., Boscaiu M., Vicente O., Moreno V., Rosselló J.A. (2002). Intra- and Interspecific Variation in DNA Content in *Cistus* (*Cistaceae*). Ann. Bot..

[33] Guzmán B., Vargas P. (2005). Systematics, character evolution, and biogeography of *Cistus* L. (*Cistaceae*) based on ITS, trnL-trnF, and matK sequences. Mol. Phylogenet. Evol..

[34] Civeyrel L., Leclercq J., Demoly J.-P., Agnan Y., Quèbre N., Pélissier C., Otto T. (2011). Molecular systematics, character evolution, and pollen morphology of *Cistus* and *Halimium* (*Cistaceae*). Plant Syst. Evol..

[35] Simões M.P., Madeira M., Gazarini L.C. (2009). Ability of *Cistus* L. shrubs to promote soil rehabilitation in extensive oak woodlands of Mediterranean areas. Plant Soil.

[36] Catoni R., Gratani L., Varone L. (2012). Physiological, morphological and anatomical trait variations between winter and summer leaves of *Cistus* species. Flora Morphol. Distrib. Funct. Ecol. Plants.

[37] Roy J., Sonie L. (1992). Germination and Population Dynamics of *Cistus* Species in Relation to Fire. J. Appl. Ecol..

[38] Amaral F., Nova Flora de Portugal (1971). Cont. E Açores Lisb..

[39] Pawluczyk M., Weiss J., Vicente-Colomer M.J., Egea-Cortines M. (2011). Two alleles of rpoB and rpoC1 distinguish an endemic European population from *Cistus heterophyllus* and its putative hybrid (*C. clausonis*) with *C. albidus*. Plant Syst. Evol..

[40] Guimarães R., Barros L., Carvalho A.M., Sousa M.J., Morais J.S., Ferreira I.C. (2009). Aromatic plants as a source of important phytochemicals: Vitamins, sugars and fatty acids in *Cistus ladanifer*, *Cupressus lusitanica* and *Eucalyptus gunnii* leaves. Ind. Crop. Prod..

[41] Ben Jemia M., Kchouk M.E., Senatore F., Autore G., Marzocco S., De Feo V., Bruno M. (2013). Antiproliferative activity of hexane extract from Tunisian *Cistus libanotis*, *Cistus monspeliensis* and *Cistus villosus*. Chem. Cent. J..

[42] Galmés J., Medrano H., Flexas J. (2007). Photosynthetic limitations in response to water stress and recovery in Mediterranean plants with different growth forms. New Phytol..

[43] Simões M.P., Madeira M., Gazarini L.C. (2008). The role of phenology, growth and nutrient retention during leaf fall in the competitive potential of two species of mediterranean shrubs in the context of global climate changes. Flora Morphol. Distrib. Funct. Ecol. Plants.

[44] Carlier J., Leitão J., Fonseca F. (2008). Population genetic structure of *Cistus ladanifer* L. (*Cistaceae*) and genetic differentiation from co-occurring *Cistus* species. Plant Species Biol..

[45] De Dato G.D., Micali M., Jaoudé R.A., Liberati D., De Angelis P. (2013). Earlier summer drought affects leaf functioning of the Mediterranean species *Cistus monspeliensis* L.. Environ. Exp. Bot..

[46] Preedy V.R. (2016). Essential Oils in Food Preservation, Flavor and Safety.

[47] Chaves N., Sosa T., Alías J.C., Escudero J.C. (2001). Identification and Effects of Interaction Phytotoxic Compounds from Exudate of *Cistus ladanifer* Leaves. J. Chem. Ecol..

[48] Chaves N., Sosa T., Escudero J.C. (2001). Plant growth inhibiting flavonoids in exudate of *Cistus ladanifer* and in associated soils. J. Chem. Ecol..

[49] Dixon R.A., Paiva N.L. (1995). Stress-Induced Phenylpropanoid Metabolism. Plant Cell.

[50] Saracini E., Tattini M., Traversi M.L., Vincieri F.F., Pinelli P. (2005). Simultaneous LC-DAD and LC-MS Determination of Ellagitannins, Flavonoid Glycosides, and Acyl-Glycosyl Flavonoids in *Cistus salvifolius* L. Leaves. Chromatographia.

[51] Barrajón-Catalán E., Fernández-Arroyo S., Roldán C., Guillén E., Saura D., Carretero A.S., Micol V. (2011). A systematic study of the polyphenolic composition of aqueous extracts deriving from several *Cistus* genus species: Evolutionary relationship. Phytochem. Anal..

[52] Farley R.A., McNeilly T. (2004). Diversity and divergence in *Cistus salvifolius* (L.) populations from contrasting habitats. Hereditas.

[53] Hegnauer M., Hegnauer R. (1962). Chemotaxonomie der Pflanzen: Eine Ubersicht uber die Verbreitung und die Systematische Bedeutung der Pflanzenstoffe.

[54] Teresa J.D.P., Urones J., Marcos I., Bermejo F., Basabe P. (1983). A rearranged labdane: Salmantic acid from *Cistus laurifolius*. Phytochemistry.

[55] Teresa J.D.P., Urones J.G., Marcos I.S., Barcala P.B., Garrido N.M. (1986). Diterpenoid and other components of *Cistus laurifolius*. Phytochemistry.

[56] Demetzos C., Harvala C., Philianos S.M., Skaltsounis A.L. (1990). A New Labdane-Type Diterpene and Other Compounds from the Leaves of *Cistus incanus* ssp. *creticus*. J. Nat. Prod..

[57] Demetzos C., Mitaku S., Couladis M., Harvala C., Kokkinopoulos D. (1994). Natural Metabolites of ent-13-epi-Manoyl Oxide and Other Cytotoxic Diterpenes from the Resin “LADANO” of *Cistus creticus*. Planta Med..

[58] Urones J.G., Basabe P., Marcos I.S., Jiménez A., Lithgow A.M., López M., Moro R.F., Gómez A. (1994). Ring a functionalized Neo-clerodane diterpenoids from *Cistus populifolius*. Tetrahedron.

[59] Berger S., Sicker D. (2009). Classics in Spectroscopy: Isolation and Structure Elucidation of Natural Products.

[60] Demetzos C., Mitaku S., Skaltsounis A.L., Harvala M.C.C., Libot F. (1994). Diterpene esters of malonic acid from the resin ‘Ladano’ of *Cistus creticus*. Phytochemistry.

[61] Angelopoulou D., Demetzos C., Perdetzoglou D. (2002). Diurnal and seasonal variation of the essential oil labdanes and clerodanes from *Cistus monspeliensis* L. leaves. Biochem. Syst. Ecol..

[62] Loizzo M.R., Ben Jemia M., Senatore F., Bruno M., Menichini F., Tundis R. (2013). Chemistry and functional properties in prevention of neurodegenerative disorders of five *Cistus* species essential oils. Food Chem. Toxicol..

[63] Oller-López J.L., Rodriguez R., Cuerva J.M., Oltra J.E., Bazdi B., Dahdouh A., Lamarti A., Ibn Mansour A. (2005). Composition of the Essential Oils of *Cistus ladaniferus* and *C. monspeliensis* from Morocco. J. Essent. Oil Res..

[64] Deschepper R. (2017). Variabilité de la Composition des Huiles Essentielles et Intérêt de la Notion de Chémotype en Aromathérapie. Ph.D. Thesis.

[65] Gomes P.B., Mata V.G., Rodrigues A.E. (2005). Characterization of the Portuguese-Grown *Cistus ladanifer* Essential Oil. J. Essent. Oil Res..

[66] Robles C., Bousquet-Mélou A., Garzino S., Bonin G. (2003). Comparison of essential oil composition of two varieties of *Cistus ladanifer*. Biochem. Syst. Ecol..

[67] Teixeira S., Mendes A., Alves A., Santos L. (2007). Simultaneous distillation–extraction of high-value volatile compounds from *Cistus ladanifer* L.. Anal. Chim. Acta.

[68] Rauwald H.W., Grötzinger K. (2010). Growth inhibiting activity of volatile oil from *Cistus creticus* L. against *Borrelia burgdorferi* s.s. in vitro. Pharmazie.

[69] Demetzos C., Loukis A., Spiliotis V., Zoakis N., Stratigakis N., Katerinopoulos H.E. (1995). Composition and Antimicrobial Activity of the Essential oil of *Cistus criticus* L.. J. Essent. Oil Res..

[70] Demetzos C., Katerinopoulos H., Kouvarakis A., Stratigakis N., Loukis A., Ekonomakis C., Spiliotis V., Tsaknis J. (1997). Composition and Antimicrobial Activity of the Essential Oil of *Cistus criticus* subsp. *eriocephalus*. Planta Med..

[71] Paolini J., Falchi A., Quilichini Y., Desjobert J.-M., De Cian M.-C., Varesi L., Costa J. (2009). Morphological, chemical and genetic differentiation of two subspecies of *Cistus creticus* L. (*C. creticus* subsp*. eriocephalus* and *C. creticus* subsp. corsicus). Phytochemistry.

[72] Robles C., Garzino S. (1998). Essential oil composition of *Cistus albidus* leaves. Phytochemistry.

[73] Maccioni S., Baldini R., Cioni P.L., Tebano M., Flamini G. (2006). In vivo volatiles emission and essential oils from different organs and pollen of *Cistus albidus* from Caprione (Eastern Liguria, Italy). Flavour Fragr. J..

[74] Paolini J., Tomi P., Bernardini A.-F., Bradesi P., Casanova J., Kaloustian J. (2008). Detailed analysis of the essential oil from *Cistus albidus* L. by combination of GC/RI, GC/MS and 13C-NMR spectroscopy. Nat. Prod. Res..

[75] Demetzos C., Angelopoulou D., Perdetzoglou D. (2002). A comparative study of the essential oils of *Cistus salviifolius* in several populations of Crete (Greece). Biochem. Syst. Ecol..

[76] Riehle P., Vollmer M., Rohn S. (2013). Phenolic compounds in *Cistus incanus* herbal infusions—Antioxidant capacity and thermal stability during the brewing process. Food Res. Int..

[77] Šarić A., Balog T., Sobočanec S., Kušić B., Šverko V., Rusak G., Likić S., Bubalo D., Pinto B., Reali D. (2009). Antioxidant effects of flavonoid from Croatian *Cystus incanus* L. rich bee pollen. Food Chem. Toxicol..

[78] Nicoletti M., Toniolo C., Venditti A., Bruno M., Ben Jemia M. (2014). Antioxidant activity and chemical composition of three Tunisian *Cistus*: *Cistus monspeliensis Cistus villosus* and *Cistus libanotis*. Nat. Prod. Res..

[79] Orhan N., Aslan M., Şüküroğlu M., Orhan D.D. (2013). In vivo and in vitro antidiabetic effect of *Cistus laurifolius* L. and detection of major phenolic compounds by UPLC–TOF-MS analysis. J. Ethnopharmacol..

[80] Arnold A.E., Lutzoni F. (2007). Diversity and host range of foliar fungal endophytes: Are tropical leaves biodiversity hotspots?. Ecology.

[81] Mekhfi H., El Haouari M., Legssyer A., Bnouham M., Aziz M., Atmani F., Remmal A., Ziyyat A. (2004). Platelet anti-aggregant property of some Moroccan medicinal plants. J. Ethnopharmacol..

[82] Aziz M., Tab N., Karim A., Mekhfi H., Bnouham M., Ziyyat A., Melhaoui A., Legssyer A. (2006). Relaxant effect of aqueous extract of *Cistus ladaniferus* on rodent intestinal contractions. Fitoterapia.

[83] El Kabbaoui M., Chda A., Azdad O., Mejrhit N., Aarab L., Bencheikh R., Tazi A. (2016). Evaluation of hypoglycemic and hypolipidemic activities of aqueous extract of *Cistus ladaniferus* in streptozotocin-induced diabetic rats. Asian Pac. J. Trop. Biomed..

[84] Belmokhtar M., Bouanani N.E., Ziyyat A., Mekhfi H., Bnouham M., Aziz M., Matéo P., Fischmeister R., Legssyer A. (2009). Antihypertensive and endothelium-dependent vasodilator effects of aqueous extract of *Cistus ladaniferus*. Biochem. Biophys. Res. Commun..

[85] Youbi A.E.H.E., El Mansouri L., Boukhira S., Daoudi A., Bousta D. (2016). In Vivo Anti-Inflammatory and Analgesic Effects of Aqueous Extract of *Cistus ladanifer* L. From Morocco. Am. J. Ther..

[86] Barrajón-Catalán E., Fernández-Arroyo S., Saura D., Guillén E., Fernández-Gutiérrez A., Carretero A.S., Micol V. (2010). *Cistaceae* aqueous extracts containing ellagitannins show antioxidant and antimicrobial capacity, and cytotoxic activity against human cancer cells. Food Chem. Toxicol..

[87] El Kabbaoui M., Chda A., El-Akhal J., Azdad O., Mejrhit N., Aarab L., Bencheikh R., Tazi A. (2017). Acute and sub-chronic toxicity studies of the aqueous extract from leaves of *Cistus ladaniferus* L. in mice and rats. J. Ethnopharmacol..

[88] Dimas K., Demetzos C., Angelopoulou D., Kolokouris A., Mavromoustakos T. (2000). Biological activity of myricetin and its derivatives against human leukemic cell lines in vitro. Pharmacol. Res..

[89] Demetzos C., Dimas K., Hatziantoniou S., Anastasaki T., Angelopoulou D. (2001). Cytotoxic and Anti-Inflammatory Activity of Labdane and cis-Clerodane Type Diterpenes. Planta Medica.

[90] Bouyahya A., Abrini J., El-Baabou A., Dakka Y.B.A.N. (2016). Determination of Phenol Content and Antibacterial Activity of Five Medicinal Plants Ethanolic Extracts from North-West of Morocco. J. Plant Pathol. Microbiol..

[91] Sayah K., Marmouzi I., Mrabti H.N., Cherrah Y., Faouzi M.E.A. (2017). Antioxidant Activity and Inhibitory Potential of *Cistus salviifolius* (L.) and *Cistus monspeliensis* (L.) Aerial Parts Extracts against Key Enzymes Linked to Hyperglycemia. BioMed Res. Int..

[92] Qa’Dan F., Petereit F., Mansoor K., Nahrstedt A. (2006). Antioxidant oligomeric proanthocyanidins from *Cistus salvifolius*. Nat. Prod. Res..

[93] El Euch S.K., Bouajila J., Bouzouita N. (2015). Chemical composition, biological and cytotoxic activities of *Cistus salviifolius* flower buds and leaves extracts. Ind. Crop. Prod..

[94] Sayah K., Chemlal L., Marmouzi I., El Jemli M., Cherrah Y., Faouzi M.E.A. (2017). In vivo anti-inflammatory and analgesic activities of *Cistus salviifolius* (L.) and *Cistus monspeliensis* (L.) aqueous extracts. S. Afr. J. Bot..

[95] Chiocchio I., Mandrone M., Sanna C., Maxia A., Tacchini M., Poli F. (2018). Screening of a hundred plant extracts as tyrosinase and elastase inhibitors, two enzymatic targets of cosmetic interest. Ind. Crop. Prod..

[96] Bouyahya A., Abrini J., Et-Touys A., Bakri Y., Dakka N. (2017). Indigenous knowledge of the use of medicinal plants in the North-West of Morocco and their biological activities. Eur. J. Integr. Med..

[97] López-Orenes A., Ros-Marín A.F., Ferrer M.A., Calderón A.A. (2013). Antioxidant Capacity as a Marker for Assessing the In Vitro Performance of the Endangered *Cistus heterophyllus*. Sci. World J..

[98] Hernández I., Alegre L., Munné-Bosch S. (2004). Drought-induced changes in flavonoids and other low molecular weight antioxidants in *Cistus clusii* grown under Mediterranean field conditions. Tree Physiol..

[99] Bouyahya A., Abrini J., Talbaoui A., Et-Touys A., Chatoui K., Harhar H., Bakri Y., Dakka N. (2017). Phytochemical Screening, Antiradical and Antibacterial Activities of *Cistus crispus* from Morocco. J. Mater. Environ. Sci..

[100] Bouyahya A., Bakri Y., Et-Touys A., Assemian I.C.C., Abrini J., Dakka N. (2018). *In vitro* antiproliferative activity of selected medicinal plants from the North-West of Morocco on several cancer cell lines. Eur. J. Integr. Med..

[101] Dimas K., Demetzos C., Marsellos M., Sotiriadou R., Malamas M., Kokkinopoulos D. (1998). Cytotoxic Activity of Labdane Type Diterpenes Against Human Leukemic Cell Lines in vitro. Planta Medica.

[102] Matsingou C., Hatziantoniou S., Georgopoulos A., Dimas K., Terzis A., Demetzos C. (2005). Labdane-type diterpenes: Thermal effects on phospholipid bilayers, incorporation into liposomes and biological activity. Chem. Phys. Lipids.

[103] Skorić M., Todorović S., Gligorijević N., Janković R., Živković S., Ristić M., Radulović S. (2012). Cytotoxic activity of ethanol extracts of in vitro grown *Cistus creticus* subsp. *creticus L. on human cancer cell lines*. Ind. Crop. Prod..

[104] Barkaoui M., Katiri A., Boubaker H., Msanda F. (2017). Ethnobotanical survey of medicinal plants used in the traditional treatment of diabetes in Chtouka Ait Baha and Tiznit (Western Anti-Atlas), Morocco. J. Ethnopharmacol..

[105] Manousidis T., Parissi Z., Kyriazopoulos A., Malesios C., Koutroubas S., Abas Z. (2018). Relationships among nutritive value of selected forages, diet composition and milk quality in goats grazing in a Mediterranean woody rangeland. Livest. Sci..

[106] Attaguile G., Perticone G., Mania G., Savoca F., Pennisi G., Salomone S. (2004). *Cistus incanus* and *Cistus monspeliensis* inhibit the contractile response in isolated rat smooth muscle. J. Ethnopharmacol..

[107] Hatziantoniou S., Dimas K., Georgopoulos A., Sotiriadou N., Demetzos C. (2006). Cytotoxic and antitumor activity of liposome-incorporated sclareol against cancer cell lines and human colon cancer xenografts. Pharmacol. Res..

[108] Slezak A., Moreira H., Szyjka A., Oszmianski J., Gasiorowski K. (2017). Conditions of prooxidant activity of *cistus* and pomegranate polyphenols in v79 cell cultures. Acta Pol. Pharm. Drug Res..

[109] Yeşilada E., Üstün O., Sezik E., Takaishi Y., Ono Y., Honda G. (1997). Inhibitory effects of Turkish folk remedies on inflammatory cytokines: Interleukin-1α, interleukin-1β and tumor necrosis factor α. J. Ethnopharmacol..

[110] Sadhu S.K., Okuyama E., Fujimoto H., Ishibashi M., Yesilada E. (2006). Prostaglandin inhibitory and antioxidant components of *Cistus laurifolius*, a Turkish medicinal plant. J. Ethnopharmacol..

[111] Küpeli E., Yesilada E. (2007). Flavonoids with anti-inflammatory and antinociceptive activity from *Cistus laurifolius* L. leaves through bioassay-guided procedures. J. Ethnopharmacol..

[112] Ark M., Ustun O., Yesilada E. (2004). Analgesic Activity of *Cistus laurifolius* in Mice. Pharm. Biol..

[113] Kupeli E., Orhan D.D., Yesilada E. (2006). Effect of *Cistus laurifolius* L. leaf extracts and flavonoids on acetaminophen-induced hepatotoxicity in mice. J. Ethnopharmacol..

[114] Akkol E.K., Orhan I.E., Yesilada E. (2012). Anticholinesterase and antioxidant effects of the ethanol extract, ethanol fractions and isolated flavonoids from *Cistus laurifolius* L. leaves. Food Chem..

[115] Enomoto S., Okada Y., Güvenc A., Erdurak C.S., Coşkun M., Okuyama T. (2004). Inhibitory Effect of Traditional Turkish Folk Medicines on Aldose Reductase (AR) and Hematological Activity, and on AR Inhibitory Activity of Quercetin-3-*O*-methyl Ether Isolated from *Cistus laurifolius* L.. Biol. Pharm. Bull..

[116] Papaefthimiou D., Papanikolaou A., Falara V., Givanoudi S., Kostas S., Kanellis A.K. (2014). Genus *Cistus*: A model for exploring labdane-type diterpenes’ biosynthesis and a natural source of high value products with biological, aromatic, and pharmacological properties. Front. Chem..

[117] Stępień A.E., Aebisher D., Aebisher D.B. (2018). Biological properties of *Cistus* species. Eur. J. Clin. Exp. Med..

[118] Nováková L., Pavlík J., Chrenková L., Martinec O., Červený L. (2018). Current antiviral drugs and their analysis in biological materials—Part II: Antivirals against hepatitis and HIV viruses. J. Pharm. Biomed. Anal..

[119] Chiang L., Chiang W., Chang M., Ng L., Lin C. (2002). Antiviral activity of Plantago major extracts and related compounds in vitro. Antivir. Res..

[120] Edziri H., Mastouri M., Mahjoub M.A., Ammar S., Mighri Z., Gutmann L., Aouni M. (2011). Antiviral activity of leaves extracts of Marrubium alysson L.. J. Med. Plants Res..

[121] Droebner K., Ehrhardt C., Poetter A., Ludwig S., Planz O. (2007). CYSTUS052, a polyphenol-rich plant extract, exerts anti-influenza virus activity in mice. Antivir. Res..

[122] Ehrhardt C., Hrincius E.R., Korte V., Mazur I., Droebner K., Poetter A., Dreschers S., Schmolke M., Planz O., Ludwig S. (2007). A polyphenol rich plant extract, CYSTUS052, exerts anti influenza virus activity in cell culture without toxic side effects or the tendency to induce viral resistance. Antivir. Res..

[123] Rebensburg S., Helfer M., Schneider M., Koppensteiner H., Eberle J., Schindler M., Gürtler L., Brack-Werner R. (2016). Potent in vitro antiviral activity of *Cistus incanus* extract against HIV and Filoviruses targets viral envelope proteins. Sci. Rep..

[124] Duncan C.J. (2005). What caused the Black Death?. Postgrad. Med. J..

[125] Kuchta K., Tung N.H., Ohta T., Uto T., Raekiansyah M., Grötzinger K., Rausch H., Shoyama Y., Rauwald H.W., Morita K. (2020). The old pharmaceutical oleoresin labdanum of *Cistus creticus* L. exerts pronounced *in vitro* anti-dengue virus activity. J. Ethnopharmacol..

[126] Berrin-Ozcelik O.U., Baykal T. (2016). Bioactivities of ethanolic extract and its fractions of *Cistus laurifolius* L. (*Cistaceae*) and *Salvia wiedemannii* Boiss. (*Lamiaceae*) species. Pharmacogn. Mag..

[127] Lozano R., Naghavi M., Foreman K., Lim S., Shibuya K., Aboyans V., Abraham J., Adair T., Aggarwal R., Ahn S.Y. (2012). Global and regional mortality from 235 causes of death for 20 age groups in 1990 and 2010: A systematic analysis for the Global Burden of Disease Study 2010. Lancet.

[128] Fokialakis N., Kalpoutzakis E., Tekwani B.L., Khan S.I., Kobaisy M., Skaltsounis A.L., Duke S.O. (2006). Evaluation of the antimalarial and antileishmanial activity of plants from the Greek island of Crete. J. Nat. Med..

[129] Fokialakis N., Kalpoutzakis E., Tekwani B.L., Skaltsounis A.L., Duke S.O. (2006). Antileishmanial Activity of Natural Diterpenes from *Cistus* sp. and Semisynthetic Derivatives Thereof. Biol. Pharm. Bull..

[130] Bouyahya A., Et-Touys A., Dakka N., Fellah H., Abrini J., Bakri Y. (2018). Antileishmanial potential of medicinal plant extracts from the North-West of Morocco. Beni-Suef Univ. J. Basic Appl. Sci..

[131] Bouamama H., Noel T., Villard J., Benharref A., Jana M. (2006). Antimicrobial activities of the leaf extracts of two Moroccan *Cistus* L. species. J. Ethnopharmacol..

[132] Barros L., Dueñas M., Alves C.T., Silva S., Henriques M., Santos-Buelga C., Ferreira I.C. (2013). Antifungal activity and detailed chemical characterization of *Cistus ladanifer* phenolic extracts. Ind. Crop. Prod..

[133] Roidaki A., Kollia E., Panagopoulou E., Chiou A., Varzakas T., Markaki P., Proestos C. (2016). Super foods and Super herbs: Antioxidant and Antifungal Activity. Curr. Res. Nutr. Food Sci. J..

[134] Lahcen S.A., El Hattabi L., Benkaddour R., Chahboun N., Ghanmi M., Satrani B., Tabyaoui M., Zarrouk A. (2020). Chemical composition, antioxidant, antimicrobial and antifungal activity of Moroccan *Cistus creticus* leaves. Chem. Data Collect..

[135] Ameziane N., Boubaker H., Boudyach H., Msanda F., Jilal A., Benaoumar A.A. (2007). Antifungal activity of Moroccan plants against citrus fruit pathogens. Agron. Sustain. Dev..

[136] Talibi I., Askarne L., Boubaker H., Boudyach E., Msanda F., Saadi B., Ben Aoumar A.A. (2012). Antifungal activity of some Moroccan plants against *Geotrichum candidum*, the causal agent of postharvest citrus sour rot. Crop. Prot..

[137] Karim H., Boubaker H., Askarne L., Cherifi K., Lakhtar H., Msanda F., Boudyach E., Ben Aoumar A.A. (2017). Use of *Cistus* aqueous extracts as botanical fungicides in the control of Citrus sour rot. Microb. Pathog..

[138] Karim H., Boubaker H., Askarne L., Talibi I., Msanda F., Boudyach E., Saadi B., Ben Aoumar A.A. (2016). Antifungal properties of organic extracts of eight *Cistus* L. species against postharvest citrus sour rot. Lett. Appl. Microbiol..

[139] Mrabet N., Mrabkt N., Lahlou H., Benjilali B. (1999). Effect of Moroccan *Cistus ladaniferus* L. (rockrose) extracts on the growth of four fungi. Cryptogam. Mycol..

[140] Upadhyay N., Singh V.K., Dwivedy A.K., Das S., Chaudhari A.K., Dubey N.K. (2018). *Cistus ladanifer* L. essential oil as a plant based preservative against molds infesting oil seeds, aflatoxin B1 secretion, oxidative deterioration and methylglyoxal biosynthesis. LWT.

[141] Mahmoudi H., Aouadhi C., Kaddour R., Gruber M., Zargouni H., Zaouali W., Ben Hamida N., Ben Nasri M., Ouerghi Z., Hosni K. (2016). Comparison of antioxidant and antimicrobial activities of two cultivated *Cistus* species from Tunisia. Biosci. J..

[142] Demetzos C., Stahl B., Anastassaki T., Gazouli M., Tzouvelekis L.S., Rallis M. (1999). Chemical Analysis and Antimicrobial Activity of the Resin Ladano, of its Essential Oil and of the Isolated Compounds. Planta Med..

[143] Guinoiseau E., Luciani A., Serra D.D.R., Quilichini Y., Berti L., Lorenzi V. (2015). Primary Mode of Action of *Cistus ladaniferus* L. Essential Oil Active Fractions on *Staphylococcus aureus* Strain. Adv. Microbiol..

[144] Vieira M., Bessa L.J., Martins M.R., Arantes S., Teixeira A.P.S., Mendes Â., Da Costa P.M., Belo A.D.F. (2017). Chemical Composition, Antibacterial, Antibiofilm and Synergistic Properties of Essential Oils from *Eucalyptus globulus* Labill. and Seven Mediterranean Aromatic Plants. Chem. Biodivers..

[145] Zohra M. (2011). Antibacterial activity of essential oils from *Cistus ladaniferus* L. and *Lavandula stoechas* L.. Int. J. Pharm. Res..

[146] Thielmann J., Muranyi P., Kazman P. (2019). Screening essential oils for their antimicrobial activities against the foodborne pathogenic bacteria *Escherichia coli* and *Staphylococcus aureus*. Heliyon.

[147] Kolocouris A., Mavromoustakos T., Demetzos C., Terzis A., Grdadolnik S.G. (2001). Structure elucidation and conformational properties of a novel bioactive clerodane diterpene using a combination of high field NMR spectroscopy, computational analysis and X-ray diffraction. Bioorg. Med. Chem. Lett..

[148] Móricz Á.M., Szeremeta D., Knaś M., Długosz E., Ott P.G., Kowalska T., Sajewicz M. (2018). Antibacterial potential of the *Cistus incanus* L. phenolics as studied with use of thin-layer chromatography combined with direct bioautography and in situ hydrolysis. J. Chromatogr. A.

[149] Ben Sassi A., Harzallah-Skhiri F., Aouni M. (2007). Investigation of Some Medicinal Plants from Tunisia for Antimicrobial Activities. Pharm. Biol..

[150] Güvenç A., Yildiz S., Ozkan A.M.G., Erdurak C.S., Coskun M., Yilmaz G., Okuyama T., Okada Y., Yıldız S., Yılmaz G. (2005). Antimicrobiological Studies on Turkish *Cistus.* Species. Pharm. Biol..

[151] Tomás-Menor L., Morales-Soto A., Barrajón-Catalán E., Roldan-Segura C.M., Carretero A.S., Micol V. (2013). Correlation between the antibacterial activity and the composition of extracts derived from various Spanish *Cistus* species. Food Chem. Toxicol..

[152] Bayoub K., Baibai T., Mountassif D., Retmane A. (2010). Antibacterial activities of the crude ethanol extracts of medicinal plants against *Listeria monocytogenes* and some other pathogenic strains. Afr. J. Biotechnol..

[153] Ferreira S., Santos J., Duarte A., Queiroz J., Domingues F. (2012). Screening of antimicrobial activity of *Cistus ladanifer* and *Arbutus unedo* extracts. Nat. Prod. Res..

[154] Rebaya A., Souad I., Hammrouni S., Maaroufi A., Ayadi M., Chérif J. (2016). Antibacterial and Antifungal Activities of Ethanol Extracts of *Halimium halimifolium*, *Cistus salviifolius* and *Cistus monspeliensis*. HAL.

[155] Sqalli H., El Ouarti A., Ennabili A., Ibnsouda S., Farah A., Haggoud A., Houari A., Iraqui M. (2007). Évaluation de l’effet antimycobactérien de plantes du centre-nord du Maroc. Bull. Soc. Pharm..

[156] Haouat A.C., Sqalli H., Farah A., Haggoud A., Iraqui M. (2013). Activité antimycobactérienne des extraits de deux espèces marocaines du genre *Cistus*. Phytothérapie.

[157] Rauwald H.W., Liebold T., Grötzinger K., Lehmann J., Kuchta K. (2019). Labdanum and Labdanes of *Cistus creticus* and *C. ladanifer*: Anti-Borrelia activity and its phytochemical profiling. Phytomedicine.

[158] Hannig C., Spitzmüller B., Al-Ahmad A., Hannig M. (2008). Effects of *Cistus*-tea on bacterial colonization and enzyme activities of the in situ pellicle. J. Dent..

[159] Lekbach Y., Xu D., El Abed S., Dong Y., Liu D., Khan M.S., Koraichi S.I., Yang K. (2018). Mitigation of microbiologically influenced corrosion of 304L stainless steel in the presence of *Pseudomonas aeruginosa* by *Cistus ladanifer* leaves extract. Int. Biodeterior. Biodegrad..

[160] Manikandan P. Antibacterial activities of the methanol extracts of *Cinnamomum cassia Cistus monspeliensis* and three other medicinal plants against multi-drug resistant Gram-negative bacteria. Proceedings of the National Conference on RIPP 2018.

